# A review: the botany, ethnopharmacology, phytochemistry, pharmacology of Cinnamomi cortex

**DOI:** 10.1039/d1ra04965h

**Published:** 2021-08-12

**Authors:** Songtao Liu, Liu Yang, Senwang Zheng, Ajiao Hou, Wenjing Man, Jiaxu Zhang, Song Wang, Xuejiao Wang, Huan Yu, Hai Jiang

**Affiliations:** Key Laboratory of Chinese Materia Medica, Heilongjiang University of Chinese Medicine, Ministry of Education Harbin 150040 China jianghai_777@126.com

## Abstract

Cinnamomi Cortex (CC) is the dried bark of *Cinnamomum cassia* (L.) J. Presl. Modern pharmacological research shows that CC can be used to treat diabetes, breast cancer, leukemia and other diseases. It has been used for more than 2000 years in China, mainly distributed in Guangxi, Guangdong, Yunnan and Fujian. In this paper, the botany, ethnopharmacology, phytochemistry, pharmacology, pharmacokinetics and other aspects of CC are summarized. We hope to provide convenience for the further exploration and development of CC. There are more than 300 components isolated from CC including essential oils, polyphenols, diterpenes and sesquiterpenes, flavonoids, polysaccharides and others. Pharmacological studies show that CC has a wide range of pharmacological activities such as anti-inflammatory, antibacterial, antioxidant, antitumor, improving glucose and lipid metabolism, neuroprotection and so on. It shows that CC has great potential to develop into a cheap, low-toxicity and highly-efficient natural therapeutic drug. However, there is still a long way to go for research of CC, although great progress has been made. For instance, clinical practices for CC recorded in traditional medicine books need to be paid more attention. Present achievements are still not enough to clearly explain the mechanism for some diseases. New skeletons and new drugs will be required to be discovered, so that the potential of CC can be brought into full play.

## Abstract 2

Cinnamomi Cortex (CC) is the dried bark of *Cinnamomum cassia* (L.) J. Presl.

## Graphical abstract

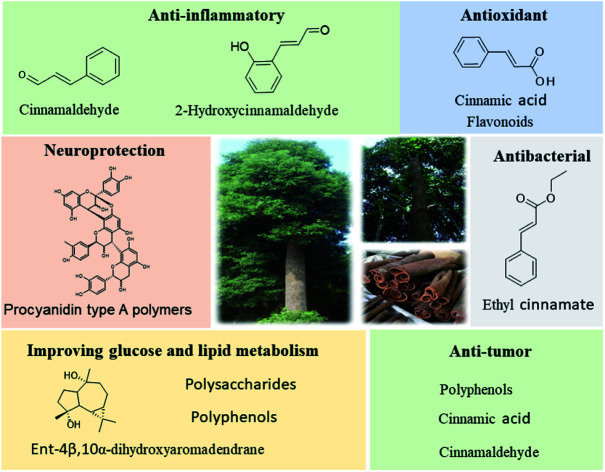

## 1. Introduction

The dried bark of *Cinnamomum cassia* (L.) J. Presl (CCP), CC is used as a food spice or medicine. CC has been cultivated and used worldwide for thousands of years. In China, people call it *rougui*. In India, CC is called *tejpat*, and it is consumed as a spice. The earliest record of *rougui* can be traced to Sheng Nong's herbal classic 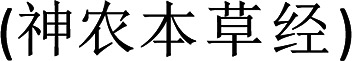. It is also listed as a top-grade herb in ancient medical volumes. The Chinese Pharmacopoeia mentions that CC is sweet and pungent in flavor; hot in property. It affects the kidney, spleen, heart, and liver meridians, where it has the functions of eliminating cold and relieving pain, promoting blood circulation and raising body temperature. Clinically, CC is used as a remedy to treat impotence, arthritis, dizziness, vomiting, fever, diarrhea, abdominal pain, cardiopathy, prostatitis, dysmenorrhea, and amenorrhea.^1^ CC is produced in Guangdong and Guangxi provinces. Phytochemical studies showed that its constituents include essential oil,^2^ polyphenols,^3^ diterpenes,^4^ flavonoids,^5^ polysaccharides,^6^ and other compounds. Pharmacological studies showed that CC's effects include anti-inflammatory,^7^ antibacterial,^8^ antioxidant,^8^ and antitumor^9^ properties; it also improves glucose and lipid metabolism,^10^ mediates neuroprotection,^11^ and performs other functions.

Despite CC's substantial therapeutic value as an herbal medicine, there is a paucity of literature, and Chinese herbal medicine books written during different dynasties have geographical and temporal differences. The same medicinal materials may have different names. The same names may represent different medicinal materials. Some studies stated that *guizhi* and *rougui* are the young branches of CCP before the Song Dynasty,^12^ while *guizhi* used in Shang Han Lun 
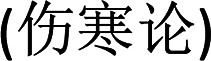
 is equivalent to the modern CC.^13^ The pharmacological effects of CC and Cinnamomi ramulus are quite different. To establish safety, it is necessary to establish a unified quality control standard. Therefore, in the present paper, botany, ethnopharmacology, phytochemistry, pharmacological effects, pharmacokinetics, and quality control of CC are comprehensively summarized. We provide a material basis for formulating a scientific herb evaluation standard to analyze the relationship between pharmacological effects and active components and suggest directions for developing new naturally-based drugs.

## 2. Botany

CC is the bark of *Cinnamomum cassia* (L.) J. Presl (CCP), which is an evergreen tree belonging to Cinnamomum Trew in Lauraceae. The trunk height is 10–15 meters. CCP is distributed in tropical and subtropical regions of Asia, including China, Vietnam, Indonesia, Srilanka, and other countries. There are 46 species and one variety in China.

**Fig. 1:**
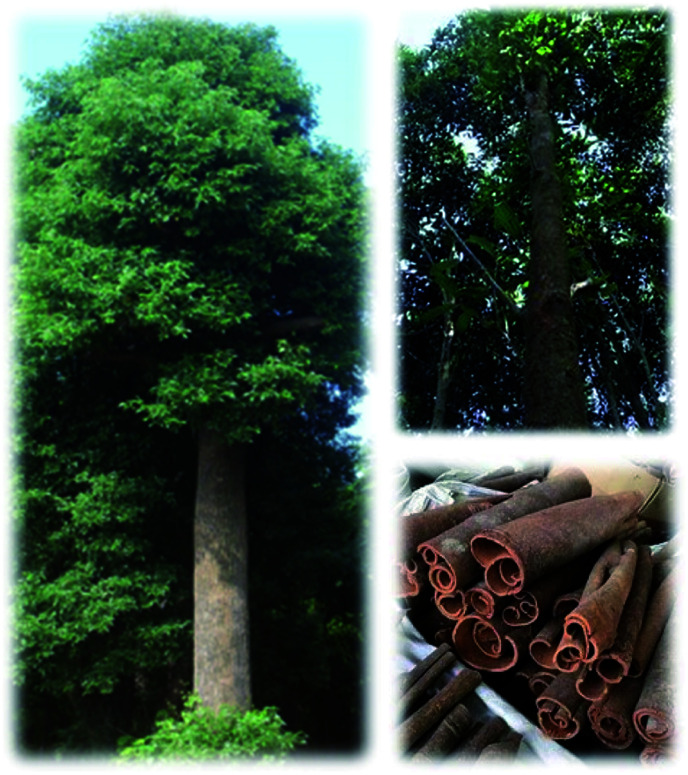
*Cinnamomum cassia* (L.) J. Presl and CC.

CCP is easy to distinguish based on appearance. The morphological characteristics are as follows: the color of the dried bark is dark brown. Its leaves alternate or are nearly opposite and are oval to nearly lanceolate in shape. They are leathery, green and shiny. The twigs are irregular quadrangles. The panicle is axillary or subterminal, with small yellow-green flowers. The fruit is oval. The flowering period is from June to August, and the fruiting period is from October to December.^14^ CC is slightly rough on one surface, with irregular longitudinal wrinkles and transverse lenticels (Fig. 1).

CC has visible white markings and thin longitudinal stripes. The inner surface is dark reddish-brown and uneven. It is easy to break and difficult to cut. Sand lines are in the middle. Slices appear semi-flaky and uneven. The outer layer is yellow-brown, rough, and granular. The inner layer is reddish-brown and oily with a faint, aromatic scent. The taste is sweet and spicy.^15^ The fibrous cells in the powder of CC are long spindle-shaped, with concave–convex edges. The cell wall is thick and shows a lignification reaction. The oily cell wall is thin and spherical. The cork cell wall is thick and polygonal. Starch granules are fine and oval.^16^

As a deep-rooted tree species, CCP grows in warm, humid, and sunny environments and is strongly shade-tolerant. It does not tolerate frost and snow, drought, stagnant water, or severe cold. It grows well in loose and fertile sandy soil that is well-drained, acidic, and rich in organic matter, including sand dunes or sloping mountains.^17^ It primarily grows in Guangdong, Guangxi, Fujian, Taiwan, and Yunnan in China and is also found in India, Laos, Vietnam, and Indonesia. Generally, the harvest time of CC is spring and autumn. People first draw a line on the tree surface, then peel off bark from the trees individually. The bark is cut into strips, putting it under the laundry basket to braise it.^18^ CC is preferred when it is exquisite, thick and heavy, unbroken, sufficiently oily, intensely fragrant, sweet and slightly pungent, with less chewing residue.

Unfortunately, herbal markets are currently full of CC imitations.^19^ It is not uncommon that businesses use other bark that has a similar appearance to CC to sell at a low price; this practice is detrimental to evaluations of quality and clinical efficacy and may even cause medical accidents. For these reasons, standardizing the quality of CC is critical. We consulted the literature and summarized the plants that are confused with CC in terms of morphological characteristics and medicinal characteristics (Table 1).

**Table 1:** The source plant and characteristics of confusions

The source plant	Characteristics		Ref.
	Morphological characteristics	Medicinal characteristics	
*Cinnamomum ponium* Sieb.	Bark is cylindrical or irregularly blocky. It is thin and dry, with light aroma, generally used as spice or seasoning	It tastes pungent and sweet, with the warm nature. And it has the effect of warming spleen and stomach for dispelling cold, regulating qi-flowing for relieving pain	100
Tian Zhu Gui			
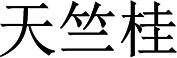			
*Cinnamomum burmanni* (Nees et T.Nees) Blume.	It is irregular pieces, thin and dry, with no yellow-brown lines between the inner and outer layers, with the light aroma and more mucus	It tastes slightly pungent and sweet, with the warm nature. And it has the effect of dispelling wind and cold, warming the baby and relieving pain	99
Yin Xiang			
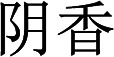			
*Cinnamomum tamala* (Ham.) Nees et Eberm.	Bark is mostly semi-cylindrical, with dark brown outer skin and gray-white pattern, dark brown inner surface and yellow-brown lines between them, contains a lot of mucus, and has a faint scent like camphor	It enters liver and spleen meridians, tastes pungent and sweet, with the warm nature. And it has the effect of warming and dredging meridians, activating qi and relieving pain	99
Chai Gui			
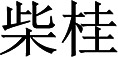			
*Lindera obtusiloba* Bl.	Its outer surface is reddish brown, with occasional gray spots, and its inner surface is reddish brown and smooth. It is easy to break, slightly fragrant, light in taste, and the water extract has more mucus	It tastes pungent, with the warm nature. And it has the effect of promoting blood circulation for removing blood stasis and detumescence	98
San Ya Wu Yao			
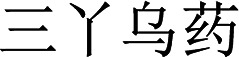			
*Machilus pauhoi* Kanehira	The outer surface is brown and smooth, the inner surface is brown, which is not easy to break, slightly fragrant, slightly bitter and astringent, and the chewing viscosity is large. After soaking in water, there is a lot of mucus on the inner surface	As an ornamental plant, it is generally not used for medicinal purposes	101
Pao Hua Nan			
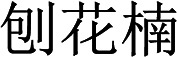			
*Cinnamomum subavenium* Miq.	It is cylindrical or irregular, with thin skin, hard texture, dry and non-oily, and light aroma	It tastes pungent, with the warm nature. And it has the effect of warming spleen and stomach for dispelling cold, regulating qi-flowing for relieving pain, promoting blood circulation for removing obstruction in collaterals	102
Xiang Gui			
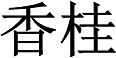			
*Cinnamomum mairei* Levl.	The outer skin is dark brown, with gray-white patterns, the inner surface is dark brown, with yellow-brown lines between them, with faint scent like camphor	It tastes pungent and sweet, with the warm nature. And it has the functions of warming channels, dispelling cold, promoting qi and blood circulation and relieving pain	102
Yin Ye Gui			
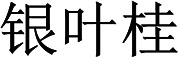			
*Litsea pungens* Hemsl.	The outer skin is dark brown, the inner surface is dark brown, with yellow-brown lines between them, with faint scent like camphor	It tastes pungent and sweet, with the warm nature. And it has the functions of stopping bleeding, set bones, clear meridians and activate collaterals	102
Mu Jiang Zi			
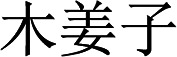			

## 3. Ethnopharmacology

### 3.1 Traditional uses

CC has been used in China for thousands of years and recorded in many Chinese medicine books. It warms the spleen and stomach, promoting blood circulation. The recommended dosage is 1–5 g. Individuals with bleeding tendency or pregnant women should use it with caution. CC cannot be used with halloysitum rubrum. The adsorption effect of halloysitum rubrum influences the dissolution of the essential oil, reducing or weakening the sedative and analgesic effects of CC.^20^ There are some records in the classic literature.

De Pei Ben Cao 
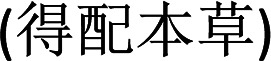
 of the Qing Dynasty recorded that sputum-producing cough and pharyngeal pain, insufficient qi and blood and excessive internal heat, pregnancy, and postpartum blood heat, were four prohibitions for CC using.

Tang Ben Cao 
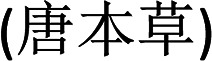
 of the Tang Dynasty wrote that CCP had two or three layers of bark, with three lines in the leaves, and the texture was as thin as bamboo. The skin of the large branches and twigs was full of laurel; however, the skin of the large branches cannot be rewound. Its taste is very mild, so it is not used as medicine. The appearance of its bark and leaves were recorded.

For Yu Qiu Yao Jie 
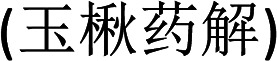
 of Qing Dynasty, CC is the bark herb, which is generally considered to treat superficial lesions. However, later studies found that it can also treat liver and kidney diseases deeply.

The medicine books of Bie Lu 
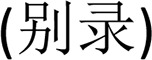
 of the Han Dynasty pointed that CC is used for angina pectoris, warming tendons, dredging veins, relieving vexation, sweating, headache, waist pain, relieving salivation and curing cough. It warms the middle, benefiting the liver and lungs. It also causes abortion, strengthens the joints, passes the blood vessels, and complements insufficiency. It illustrated the broad clinical application of CC, in which the effect of blood circulation will lead to abortion. However, doctor Pang An Shi in Song Dynasty said that frying would not damage the fetus, indicating the importance of processing herbs.

Yi Yu Lu 
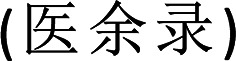
 records a particular treating case for eye inflammation with swelling and paining. Patient is anorexia with the spleen deficiency. His liver was hyperstimulation, but his spleen was fatigue. Treating the liver only with suppressive medicine will make his spleen more fatigued, while treating the spleen only with warm medicine will make the liver more stimulating. Surprisingly, adding CC into the mild medicine can both inhibit the liver and benefit the spleen. It successfully treats two symptoms with one herb.

### 3.2 Compound compatibility and their uses

CC plays an essential therapeutic role in combination with other drugs. There are lots of compound preparations in classical Chinese medical monographs. These prescriptions comply with the theory of “*Jun Chen Zuo Shi*.”^21^ The “*Jun*” drugs can treat major diseases, the “*Chen*” drugs can enhance the efficacy of the monarch drugs, the “*Zun*” drugs can eliminate the toxic and side effects of herbs, and the “*Shi*” drugs can promote the absorption of drugs in the body.^96^ The efficacy of prescriptions was not only exerted by a single way, but through multi-channel, multi-target synergy or antagonism to achieve the ultimate therapeutic goals.^148^ The herb pairs can keep the health of the human body through the synergistic effect of herbs and have reputed effectiveness and a relative absence of side effects. By comparison, chemotherapeutic drugs are targeted and quickly, but they could produce many side effects, such as myelosuppression, decreased immune function, organ damage, hair loss and so on.^149^

In clinic practice, CC can be used by combining with other herbs.

CC combined with white raisins, gypsum, Carthami flos, Glycyrrhizae radix et rhizoma, Cyperi rhizoma, pomegranate, used for clearing lung heat, relieving cough, senile asthma, chest fullness, and depression.

CC combined with Rehmanniae radix praeparata, radix aconiti lateralis preparata slice, Moutan cortex, Achyranthis bidentatae radix, and Psoraleae fructus, Amomi fructus, Plantaginis semen, *Alpinia oxyphylla*, Dioscoreae rhizoma, Alismatis rhizoma, Rosae Laevigatae fructus, used for warming kidney, inspiring spleen, and eliminating phlegm. They also treat cough and asthma caused by deficiency of lung and spleen and can prevent relapse of chronic asthma.

CC combined with Coptidis rhizoma can treat insomnia caused by disorder of physiological coordination between heart and kidney.

CC is compatible with Caryophylli flos. Piperis Longi fructus protects the spleen and relieves cold and diarrhea. It is also suitable for the auxiliary treatment of diarrhea and abdominal pain in children. The commonly used formulations of CC include the *Shiliuwei Dongqing* pill, *Fugui Gutong* tablet, and *Tianhe zhuifeng* plaster. There are also *Huixiang Juhe* pill and *Anmo Ruan* ointment, used to promote blood circulation, reduce swelling, and relieve pain, hernia, and testicular swelling and pain. Others promote digestion, remove phlegm, break nodules, strengthen the body, and be used for dyspepsia, gastric distention and stomach and liver discomfort caused by sudden phlegm disease and epigastric pain. The dosage forms include decoction, tablet, capsule, granule, pill, plaster, powder, ointment, and others. Common compatibilities and clinical applications of CC from the Chinese pharmacopeia or classic Chinese medicine books are summarized in Table 2.

**Table 2:** Traditional and clinical uses of Cinnamomi cortex in China

Preparation name	Main compositions	Formulation	Traditional and clinical uses	Reference
Wenweishu Jiaonang	Cinnamomi cortex 90 g, Amomi fructus 60 g, Citri reticulatae Pericarpium 150 g, *etc*	Capsule	Warming the middle warmer, nourishing the stomach, promoting qi circulation and relieving pain are used for stomachache caused by deficiency cold of the middle warmer. The symptoms include epigastric cold pain, abdominal distention and belching, poor appetite, aversion to cold and weakness, chronic atrophic gastritis and superficial gastritis	Chinese Pharmacopoeia^1^
Qiwei Putao san	Cinnamomi cortex 60 g, Cyperi rhizoma 60 g, pomegranate 60 g, *etc*	Powder	Clearing lung heat, relieving cough, and relieving asthma are used for cough due to asthenia, senile asthma, and chest fullness and depression	Chinese Pharmacopoeia^1^
Pazhu Wan	Cinnamomi cortex 80 g, Amomi fructus rotundus 40 g, Piperis longi fructus 40 g, *etc*	Pill	Promoting digestion and dispelling cold, removes phlegm, breaks lumps, nourishes glory and strengthens body, and also used for dyspepsia, gastric distention, gastric pantothenic acid fever, and gastric liver discomfort caused by Jian Tu phlegm disease and gastric lumps and lumps	Chinese Pharmacopoeia^1^
Di Huang Rou Hui Tang	Cinnamomi cortex, Codonopsis radix, Rehmanniae radix Praeparata, Pinelliae rhizoma, *Angelicae sinensis* radix et rhizoma, Euodiae fructus	Decoction	Treatment of heat supplement and main nausea, disease in the lower coke, vomit at dusk, vomit at dusk, eat for a long time and then go out	Bu Zhi Yi Bi Yao 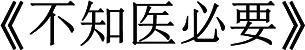 ^142^
Gui Fu Wan	Cinnamomi cortex, Aconiti Lateralis Radix Praeparata, Zingiberis rhizoma, Halloysitum rubrum	Pill	Treating diarrhea and water conservancy for a long time	Sheng Ji Zong Lu 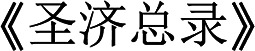 ^143^
Gui Gan Wan	Cinnamomi cortex, Rooster liver	Pill	Treating infantile enuresis during sleep	Wan Bing Hui Chun 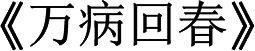 ^144^
Gui Xin Tang	Cinnamomi cortex, Glycyrrhizae radix et rhizoma, White Honey, Zingiberis rhizoma, *Angelicae sinensis* radix, Halloysitum rubrum, Aconiti lateralis radix praeparata	Decoction	It can be used for treating puerperal residual cold, dysentery, pus, blood, red and white, dozens of lines per day, and bleeding during abdominal pain	Sheng Ji Zong Lu 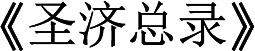 ^145^
Jiao Tai Wan	Cinnamomi cortex, Coptidis rhizoma	Pill	Treating insomnia caused by heart–kidney incompatibility	Han Shi Yi Tong 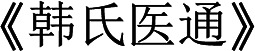 ^146^
Yang He Tang	Cinnamomi cortex, Rehmanniae radix praeparata, Ephedrae herba, Cervi cornuscolla, ginger charcoal, Glycyrrhizae radix et rhizoma	Decoction	Treating knee wind, sticking bone gangrene and all yin gangrene	Wai Ke Quan Sheng Ji 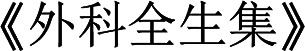 ^147^

CC of the same origin can be a medicine or food and is popular in all walks of life in China. CC is often added to foods and cakes in the food processing industry and drinks as flavoring agents and preservatives. The famous condiment “Thirteen Fragrances” contains CC. CC is also used in cosmetics, perfumes, and tobacco. There are health drinks such as CC wine and tea. CC has also been used in toothpaste and chewing gum because of its bactericidal effects.

## 4. Phytochemistry

CC contains various chemical components, including essential oil, diterpenes, sesquiterpenes, flavonoids, polyphenols, polysaccharides, and other components. In this section, the chemical constituents of CC are summarized.

### 4.1 Essential oil

**Fig. 2:**
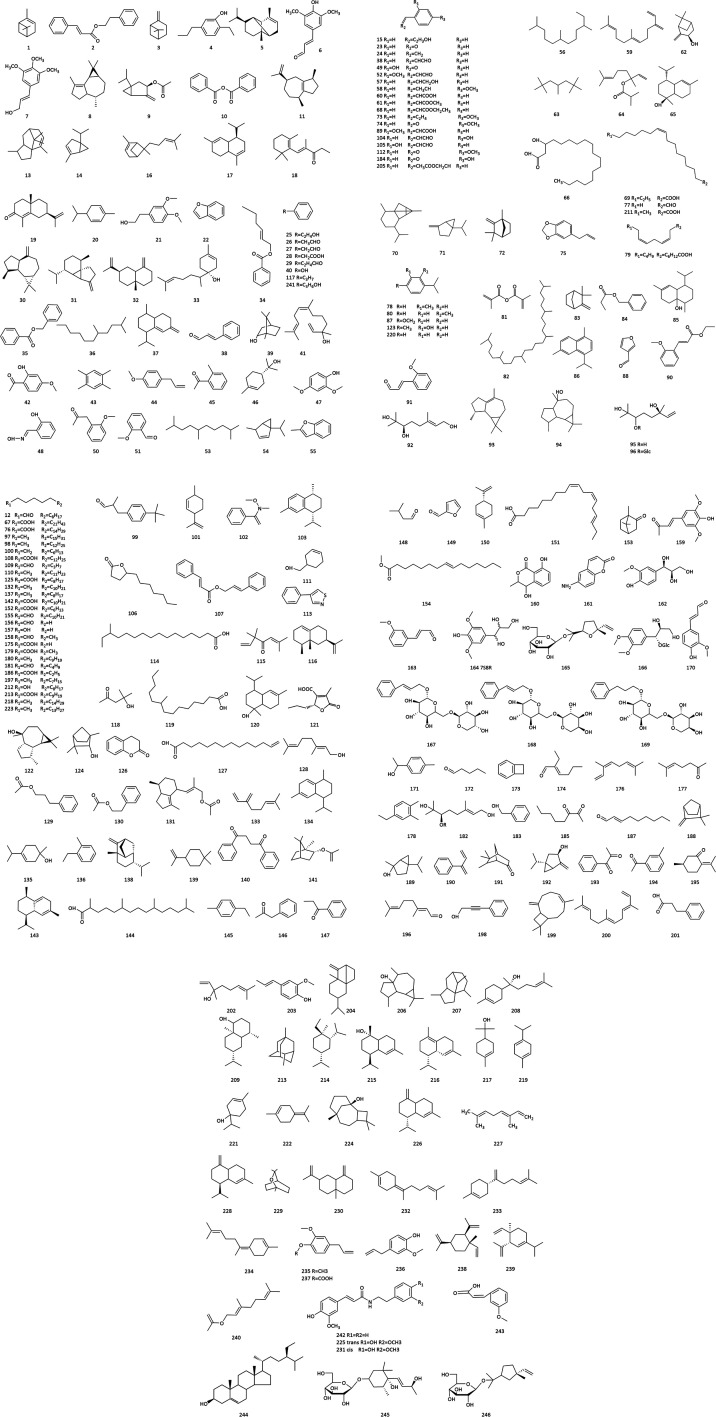
The structures of essential oils from Cinnamomi cortex.

Essential oil is the main volatile component in CC, which is a mixture with an aromatic scent. It is insoluble in water and can be extracted from plants by steam distillation. The Chinese Pharmacopoeia (2020 edition) stipulates that essential oil content should not be less than 1.2% (mL g^−1^). We searched the literature on CC from 1987 to 2021 and found that the most often mentioned components are essential oil. Therefore, we summarized the essential oil constituents in Table 3. Structures of components are shown in Fig. 2. To date, more than 200 essential oil components have been isolated and identified. Various parts of CC contain essential oil; however, the content differs substantially.^22^ Cinnamaldehyde is abundant, with a relative percentage of 76–86%.^23^ Tao *et al.* extracted 47 essential oils from the dried bark and branch bark of the CCP, which were as old as ten years, using supercritical CO_2_ extraction. The relative content of cinnamaldehyde was 90.74%.^24^ Huang *et al.* separated 60 components from dried bark and branch bark of CCP of various ages using gas chromatography-tandem mass spectrometry (GC-MS); 53 components were identified, including myrcene, *trans*-anethole, hexadecanoic acid, and others.^25^ Fenchone and α-bergamotene were first identified in CC by Bao *et al.*^26^ Chen *et al.* isolated 22 chemical constituents from an ethanolic extract of CC, and 4-hydroxymellein was isolated for the first time.^27^ Dong *et al.* identified 35 essential oil compounds from CC using GC-MS, and 21 substances were isolated for the first time.^28^ Song *et al.* extracted 54 and 56 aroma components from Ceylon cinnamon bark oil and Chinese cinnamon bark oil, respectively, using gas chromatography-time-of-flight mass spectrometry. There were some differences in the composition and content of aroma components between them.^29^ In summary, there are substantial differences in the composition of essential oil compounds in different parts of CCP. This section summarizes the components isolated from CC to provide scientific evidence for Chinese medicine theory, stating that CC is hot and Cinnamomi ramulus is warm.

**Table 3:** The essential oil components isolated from Cinnamomi cortex

No.	Components	Sources	Ref.
1	α-Pinene	CC	104
2	β-Phenethyl cinnamate	Stem bark of CCP	105
3	β-Pinene	CC	109
4	2-Ethyl-5-propylphenol	Stem bark of CCP	105
5	Ylangene	CC	107
6	Sinapaldehyde	Stem bark of CCP	125
7	*trans*-3,4,5-Trimethoxycinnamic alcohol	Stem bark of CCP	125
8	(−)-Alpha-Gurjunene	CC, stem bark of CCP	25
9	Sabinylacetate	CC	110
10	Benzoylbenzoate	Stem bark of CCP	108
11	α-Guaiene	CC	31
12	Tetradecanal	Stem bark of CCP	108
13	α-Patchoulene	CC	31
14	α-Thugene	CC	111
15	*E*-Cinnamyl alcohol	Stem bark of CCP	108
16	*trans*-α-Bergamotene	CC	107
17	α-Amorphene	CC	107
18	Dimethylionone	CC	113
19	α-Cyperone	CC	113
20	α-Phellandrene	CC	104
21	3,4-Dimethoxyphenethyl alcohol	Stem bark of CCP	105
22	Coumarone	CC	114
23	Benzaldehyde	CC	109
24	Styrene	Stem bark of CCP	112
25	Phenethyl alcohol	Stem bark of CCP	105
26	Acetophenone	CC	109
27	Benzene acetaldehyde	CC	109
28	Benzeneacetic acid	CC, stem bark of CCP, leaves of CCP	115
29	Benzenepropanal	CC	109
30	Alloaromadendrene	CC, stem bark of CCP	25
31	β-Cubebene	CC, stem bark of CCP	25
32	β-Selinene	CC, stem bark of CCP	25
33	β-Bisabolol	CC	116
34	*E*-2-Hexenyl benzoate	Leaves of CCP	23
35	Benzyl benzoate	CC	109
36	2,5-Dimethylundecane	Stem bark of CCP	105
37	(+)-*epi*-Bicyclosesquiphellandrene	CC	31
38	*trans*-Cinnamaldehyde	CC	107
39	Borneol	CC	109
40	Phenol	CC, stem bark of CCP, leaves of CCP	115
41	*trans*-Nerolidol	CC	107
42	Paeonol	Stem bark of CCP	112
43	Durene	Leaves of CCP	34
44	*P*-Allylanisole	Leaves of CCP	23
45	2-Methylacetophenone	CC, stem bark of CCP	25
46	(−)-α-Terpineol	Stem bark of CCP	138
47	2,4-Dimethoxyphenol	CC, stem bark of CCP, leaves of CCP	115
48	2-Hydroxybenzaldehyde oxime	Leaves of CCP	117
49	2-Hydroxybenzaldehyde	Stem bark of CCP	108
50	2-Methoxyphenylacetone	Leaves of CCP	117
51	2-Methoxy benzaldehyde	Leaves of CCP	23
52	2-Methoxy cinnamaldehyde	CC	123
53	2,5,9-Trimethyldecane	Stem bark of CCP	105
54	2-Thujene	Stem bark of CCP	108
55	2-Methyl benzofuran	CC	109
56	2,6,10-Trimethyl dodecane	CC	114
57	Cinnamyl alcohol	CC	123
58	*trans*-Anethole	CC, stem bark of CCP	25
59	*trans*-β-Farnesene	CC	107
60	Cinnamic acid	CC, stem bark of CCP	54
61	*trans*-Methyl cinnamate	Stem bark of CCP	112
62	(−)-*trans*-Pinocarveol	Leaves of CCP	110
63	2,2,4,6,6-Pentamethylheptane	Stem bark of CCP	105
64	Linalyl isobutyrate	Stem bark of CCP	118
65	Torreyol	CC	113
66	3-Hydroxyoctadecanoic acid	CC	119
67	Heptacosanoic acid	CC	119
68	Ethyl cinnamate	CC, stem bark of CCP,Leaves of CCP	115
69	9-Octadecenoic acid	Fruits of CCP	127
70	(+)-Cycloisosativene	CC	107
71	Sabinene	CC	111
72	(−)-Fenchone	CC	26
73	*cis*-Anethol	CC	26
74	Anisaldehyde	CC	116
75	Safrole	Stem bark of CCP	108
76	Arachidic acid	CC	119
77	(*Z*)-Hexadec-9-enal	CC	31
78	*M*-Cymene	CC	111
79	9,12-Octadecadienoic acid (*Z,Z*)-	Fruits of CCP	127
80	*O*-Cymene	Stem bark of CCP	112
81	Methacrylic anhydride	Stem bark of CCP	112
82	Squalane	CC	114
83	Camphene	CC	109
84	Benzyl propionate	Stem bark of CCP	112
85	Cubenol	CC	107
86	Cadalene	CC	106
87	Cuminaldehyde	Stem bark of CCP	118
88	3-Furaldehyde	CC	109
89	*O*-Methoxy cinnamic acid	CC	123
90	Ethyl methoxycinnamate	CC	119
91	*O*-Methoxycinnamaldehyde	CC	145
92	*trans*-Cinnamaldehyde	CC	140
93	(+)-Ledene	CC	107
94	Globulol	CC	107
95	3,7-Dimethyl-1-octene-3,6,7-triol	Immature buds of CCP	137
96	3,7-Dimethyl-oct-1-en-3,6,7-triol-6-*O*-β-d-glucopyranoside	Immature buds of CCP	137
97	Heneicosane	CC	114
98	Octadecane	CC, stem bark of CCP, leaves of CCP	110
99	Lily aldehyde	CC	124
100	Dodecane	CC	104
101	Limonene	CC	109
102	*N*-Methoxy-*N*-methylbenzamide	CR	112
103	1*S-cis*-Calamenene	CC	113
104	*P*-Hydroxycinnamaldehyde	Stem bark of CCP	118
105	2-Hydroxycinnamaldehyde	CC	123
106	Undecan-4-olide	CC	31
107	Cinnamyl cinnamate	Stem bark of CCP	22
108	Stearic acid	CC, stem bark of CCP	25
109	1-Nonanal	Stem bark of CCP	118
110	*N*-Heptadecane	CC	114
111	3-Cyclohexene-1-methanol	Fruits of CCP	127
112	*P*-Anisaldehyde	CC	114
113	4-Phenylisothiazole	CC	119
114	14-Methyl hexadecanoic acid	CC	119
115	Artemisia ketone	CR	112
116	Eremophilene	CC	106
117	Propylbenzene	CC	107
118	4-Hydroxy-4-methyl-2-pentanone	CC, stem bark of CCP	126
119	9-Methyltetradecanoic acid	CC	119
120	*T*-Cadinol	CC	107
121	Decumbic acid	CC	27
122	Viridiflorol	CC, stem bark of CCP	25
123	Carvacrol	CC	119
124	Fenchol	CC	104
125	Myristic acid	CC	119
126	Coumarin	CC, stem bark of CCP	54
127	13-Tetradecenoic acid	CC	119
128	Geraniol	Stem bark of CCP, leaves of CCP, roots bark of CCP	118
129	Phenylpropyl acetate	CC	119
130	Phenethyl acetate	Leaves of CCP	40
131	*cis*-Valerenyl acetate	CC	113
132	*N*-Hexadecane	CC	114
133	Myrcene	CC, stem bark of CCP	25
134	Cadina-1,3,5-triene	CC	120
135	1-Terpineol	Leaves of CCP	139
136	1-Ethyl-2-methyl-benzene	CC	107
137	Tetradecane	CC	114
138	(−)-Isosativene	CC	31
139	*cis*-β-Terpineol	Leaves of CCP	139
140	1,2-Dibenzoylethane	Leaves of CCP, CC, stem bark of CCP	121
141	Bornyl acetate	CC	111
142	*N*-Hexadecanoic acid	Fruits of CCP	127
143	Cadina-1,4-diene	CC	113
144	Phytol	Stem bark of CCP	22
145	1-Ethyl-4-methyl-benzene	CC	107
146	2-Propanone,1-phenyl	CC, stem bark of CCP, leaves of CCP	115
147	1-Propanone,1-phenyl	CC, stem bark of CCP, leaves of CCP	115
148	Isovaleral	Leaves of CCP	40
149	Furfural	CC	104
150	(+)-Dipentene	Leaves of CCP	40
151	Linolenic acid	CC	119
152	Dodecanoic acid	Fruits of CCP	127
153	Camphor	CC	104
154	Palmitelaidic acid methyl ester	CC	119
155	Palmitaldehyde	CC	119
156	Hexanal	CC	109
157	1-Hexanol	Leaves of CCP	40
158	Octanal	Leaves of CCP	40
159	Sinapaldehyde	CC	27
160	4-Hydroxymellein	CC	27
161	6-Aminocoumarin hydrochloride	CC	27
162	Erythro-guaiacylglycerol	CC	35
163	(*E*)-3-(3-Methoxyphenyl) acrylaldehyde	CC	35
164	(7*R*,8*S*)-Syringoylglycerole	CC	35
165	(7*S*,8*S*)-Syringoylglycerole	CC	35
166	4-Methoxy guaiacylglycerol-7-*O*-β-d-glucopyrranosiae	CC	35
167	Rosavin	Stem bark of CCP	122
168	Cinnamyl	CC	35
169	2-Phenylethyl-1-*O*-α-L-arabinopyranosy (1→6)-β-d-glucopyranoside	CC	35
170	Coniferaldehyde	CC	123
171	4-Dimethyl-alpha-benzenemethanol	CC	104
172	Pentanal	CC	109
173	Cardene	CC	109
174	2-Ethyl-2-hexenal	CC	109
175	Hexanoic acid	CC	109
176	(*Z*)-beta-Ocimene	CC	109
177	Methyl heptenone	CC	109
178	4-Ethyl-*O*-xylene	CC	109
179	Heptanoic acid	CC	109
180	*N*-Pentadecane	CC	114
181	Decanal	CC	109
182	(6*R*)-Geraniol-6,7-diol	Immature buds of CCP	137
183	Benzyl alcohol	CC	109
184	*P*-Hydroxy-benzaldehyde	CC	109
185	2,3-Octanedione	CC	109
186	Octanoic acid	CC	109
187	2-Nonenal	CC	109
188	Camphenehydrate	CC	109
189	iso-Thujol	CC	109
190	2-Phenyl-1,3-butadiene	CC	109
191	*exo*-Isocamphanone	CC	109
192	Sabinol	CC	109
193	1-Phenyl-1,2-propanedione	CC	109
194	*M*-Methylacetophenone	CC	109
195	Pulegone	CC	109
196	Citral	CC	109
197	*N*-Tridecane	CC	114
198	3-Phenyl-2-propyn-1-ol	CC	109
199	Isocaryophellene	CC	109
200	Farnesene	CC	109
201	Benzenepropionic acid	CC	109
202	Linalool	CC	140
203	Isoeugenol	CC	109
204	(+)-Sativen	CC	109
205	Cinnamyl acetate	CC	109
206	Palustrol	CC	109
207	Patchulane	CC	109
208	α-Bisabolol	CC	109
209	Eudesmol	CC	109
210	Pentadecanoic acid	CC	109
211	9-Hexadecenoic acid	CC	109
212	7-Tetradecen-1-ol	CC	109
213	*cis*-1,4-Dimethyladamantane	CC	104
214	Γ-Elemane	CC	104
215	δ-Cadinol	CC	124
216	δ-Amorphene	Stem bark of CCP	108
217	α-Terpineol	CC, stem bark of CCP	25
218	*N*-Eicosane	CC	114
219	Γ-Terpinene	CC	109
220	*P*-Cimene	Stem bark of CCP	108
221	4-Terpineol	CC	109
222	Terpinolene	CC	109
223	*N*-Nonadecane	CC	114
224	Caryophyllene alcohol	CC	107
225	*N-trans*-Feruloylmethoxytyramine	Stem bark of CCP	125
226	Γ-Muurolene	CC	107
227	*E*-β-Ocimene	Stem bark of CCP	108
228	γ-Cadinene	CC	107
229	*P*-Cineole	CC	109
230	Eudesma-4(14),11-diene	CC	120
231	*N-cis*-Feruloylmethoxytyramine	Stem bark of CCP	125
232	*Trans*-α-bisabolene	CC	107
233	β-Bisabolene	CC	106
234	*trans*-γ-Bisabolene	CC, stem bark of CCP	25
235	Methyl eugenol	CC	111
236	Eugenol	Stem bark of CCP	108
237	Eugenyl acetate	Stem bark of CCP	108
238	(−)-β-Elemene	CC	109
239	δ-Elemene	CC	113
240	Geranyl acetate	CC	140
241	Hydrocinnamic alcohol	Stem bark of CCP	108
242	(*E*)-3-(4-Hydroxy-3-methoxyphenyl)-*N*-phenthylacrylamide	Stem bark of CCP	125
243	*cis*-2-Methoxycinnamic acid	CC, stem bark of CCP	145
244	β-Sitosterol	CC	27
245	(3*S*,5*R*,6*S*,9*S*)-3,6,9-Trihydroxymegastigman-7-ene 3-*O*-β-d-glucopyranoside	Immature buds of CCP	137
246	*trans*-Linalool-3,6-oxide-β-d-glucopyranoside	Immature buds of CCP	137

Modern pharmacological research showed that essential oil has analgesic, spasmolytic, and antipyretic effects; it also lowers blood pressure, has antibacterial properties, increases leukocyte counts, combats tumors and ulcers, strengthens yang, among other effects.^30^ The differences in essential oil components and contents may be caused by different habitats, different parts, and different harvest years and extraction methods. Therefore, it is essential to establish unified quality evaluation standards to conduct in-depth compound studies of CC.

### 4.2 Diterpenoids and sesquiterpenoids

**Fig. 3:**
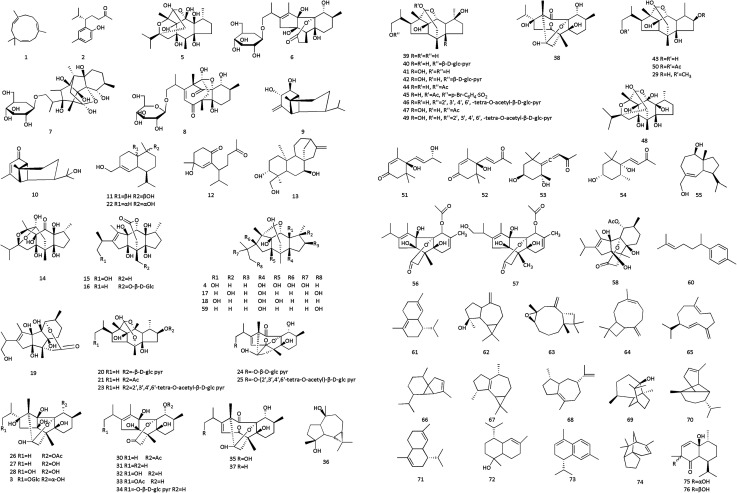
Structures of diterpenoids and sesquiterpenoids from Cinnamomi cortex.

Renoalkanes diterpenoids are distinct components of CC. Their basic structure is a pentacyclic (O) ring system. The 1-position hydroxyl group can be acetylated, oxidized, or isomerized, the 2, 9, 12, and 18-positions are substituted by methyl groups, the 5, 7, 8, and 13-positions are substituted by polyhydroxyl groups, the 11-position is a hemiacetal structure, and a hydroxyl group or glycoside can substitute the 19-position. The renoalkanes diterpenoids isolated from CC are as follows: cinncassiols A, B, C1, C2, C3, D1, D2, D3, D4, G1, α-caryophyllene, cinncassiol A-19-*O*-β-d-glucopyranoside, cinncassiol B-19-*O*-β-d-glucopyranoside, cinncassiol C1-19-*O*-β-d-glucopyranoside, cinncassiol C1-glucopyranoside, cinncassiol D1-glucopyranoside, cinncassiol D2-glucopyranoside, cinncassiol D4-glucopyranoside, 4-hydroxy-1,10-seco-muurol-5-ene-1,10-dione and so on.^27,31–36^ We summarized terpenoids and sesquiterpenoids from the literature in Table 4, and the structures of components are shown in Fig. 3.

**Table 4:** Diterpenoids and sesquiterpenoids isolated from Cinnamomi cortex

No.	Components	Sources	Ref.
1	α-Caryophyllene	CC	31
2	Litseachromolaevane A	CC	27
3	Cinncassiol B glucoside	CC	32
4	Cinncassiol D4	CC	32
5	Cinncassiol E	CC	32
6	Cinncassiol A-19-*O*-β-d-glucopyranoside	CC	33
7	Cinncassiol B-19-*O*-β-d-glucopyranoside	CC	33
8	Cinncassiol C1-19-*O*-β-d-glucopyranoside	Leaves of CCP	129
9	Cinnamoid D	CC	103
10	Cinnamoid E	CC	103
11	(−)-15-Hydroxy-T-muurolol	CC	103
12	4-Hydroxy-1,10-seco-muurol-5-ene-1,10-dione	CC	27
13	Foliol	Leaves of CCP	118
14	Cinncassiol F	CC	35
15	Cinncassiol G	CC	35
16	Cinncassiol 16-*O*-β-d-glucopyranoside-19-deoxycinncassiol G	CC	95
17	18-Hydroxyperseanol	CC	35
18	Perseanol	CC	35
19	Cinnacasol	CC	35
20	Cinncassiol D4 glucoside	CC	128
21	Cinncassiol D4-2-*O*-monoacetate	CC	128
22	15-Hydroxy-α-cadinol	CC	103
23	2′,3′,4′,6′-Tetraacetyl cinncassiol D4 glucoside	CC	128
24	Cinncassiol C1 glucoside	CC	129
25	Cinncassiol C1 19-*O*-(2′,3′,4′,6′-tetra-o-acetyl)-β-d-glucopyranoside	CC	129
26	Cinnzeylanine	CC	36
27	Cinnzeylanol	CC	36
28	Cinncassiol B	CC	36
29	Cinncassiol D3-19-*O*-monomethyl ether	CC	36
30	Anhydrocinnzeylanine	CC	36
31	Anhydrocinnzeylanol	CC	36
32	Cinncassiol A	CC	36
33	Cinncassiol A monoacetate	CC	36
34	Cinncassiol A glucoside	CC	36
35	Cinncassiol C1	CC	36
36	*ent*-4β,10α-Dihydroxyaromadendrane	CC	103
37	Cinncassiol C2	CC	36
38	Cinncassiol C3	CC	36
39	Cinncassiol D1	CC	36 and 130
40	Cinncassiol D1 glucoside	CC	36 and 130
41	Cinncassiol D2	CC	36
42	Cinncassiol D2 glucoside	CC	36
43	Cinncassiol D3	CC	36
44	Cinncassiol D1-19-*O*-monoacetate	CC	36
45	11-Monoacetyl cinncassiol D1-19-*O*-monobrosylate	CC	36
46	2′,3′,4′,6′-Tetra-*O*-acetyl cinncassiol D1 glucoside	CC	36
47	Cinncassiol D2-19-*O*-monoacetate	CC	36
48	1,4-Cyclic carbonate	CC	36
49	2′,3′,4′,6′-Tetra-*O*-acetyl cinncassiol D2 glucoside	CC	36
50	Cinncassiol D3-2-19-diacetate	CC	36
51	Blumenol A	Stem bark of CCP	125
52	Dehydrovomifoliol	Stem bark of CCP	125
53	Grasshopper ketone	Stem bark of CCP	125
54	Boscialin	Stem bark of CCP	125
55	1-(3-Indolyl)-2,3dihydroxypropan-1-one	Stem bark of CCP	125
56	2,3-Dehydroanhydrocinnzeylanine	CC	97
57	1-Acetylcinncassiol A	CC	97
58	18-Hydroxycinnzeylanine	CC	97
59	Cinncassiol D3 glucoside	CC	95
60	Curcumene	Stem bark of CCP	138
61	δ-Cadinene	CC	106
62	Espatulenol	Stem bark of CCP	138
63	Caryophyllene oxide	CC	107
64	*trans*-Caryophyllene	CC, stem bark of CCP	25
65	Germacrene D	CC	109
66	α-Cubebene	Stem bark of CCP	112
67	(−)-Isoledene	CC	104
68	α-Bulnesene	CC	141
69	Patchouli alcohol	CC	141
70	α-Copaene	Stem bark of CCP	108
71	α-Muurolene	CC	106
72	(−)-alpha-Cadinol	CC, stem bark of CCP	138 and 141
73	α-Calacorene	CC	106
74	α-Cedrene	CC	109
75	Cinnamoid A	CC	103
76	Cinnamoid B	CC	103

### 4.3 Flavonoids

**Fig. 4:**
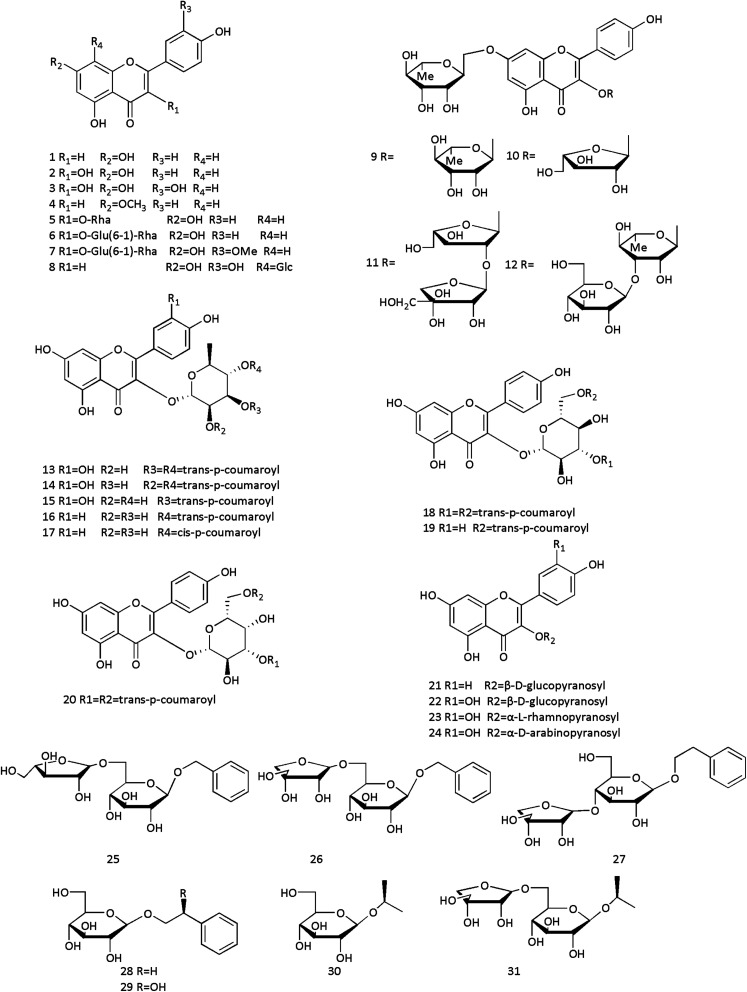
Structures of flavonoids from Cinnamomi cortex.

CC also contains flavonoids. Flavonoids are compounds with a 2-phenylchromone as the mother nucleus and C6–C3–C6 as the basic carbon skeleton. They can detoxify, antibacterial and anti-inflammatory, also possess antitumor activity and improve immunity. Mei *et al.* extracted five flavonoids from the ethanolic extract of aerial parts of CCP, including kaempferol, kaempferol-3-*O*-α-l-rhamnoside, kaempferol-3-*O*-rutinoside, isorhamnetin-3-*O*-rutinoside, and orientin^37^ In addition, there are also have apigenin, quercetin, genkwanin, 3-*O*-α-l-rhamnopyranosyl kaempferol 7-*O*-α-l-rhamnopyranoside, 3-*O*-α-l-arabinofuranosyl kaempferol 7-*O*-α-l-rhamnopyranoside, 3-*O*-β-d-apiofuranosyl-(1→2)-α-l-arabinofuranosyl kaempferol 7-*O*-α-l-rhamnopyranoside, 3-*O*-β-d-glucopyranosyl-(1→3)-α-l-rhamnopyranosyl kaempferol 7-*O*-α-l-rhamnopyranoside and so on.^38,39^ We summarize the flavonoids in Table 5 and the structures of components in Fig. 4.

**Table 5:** Flavonoids isolated from Cinnamomi cortex

No.	Components	Sources	Ref.
1	Apigenin	Stem bark of CCP	38
2	Kaempferol	Stem bark of CCP	38
3	Quercetin	Stem bark of CCP	38
4	Genkwanin	Stem bark of CCP	38
5	Kaempferol-3-*O*-α-l-rhamnoside	Stem bark of CCP	37
6	Kaempferol-3-*O*-rutinoside	Stem bark of CCP	37
7	Isorhamnetin-3-*O*-rutinoside	Stem bark of CCP	37
8	Orientin	Stem bark of CCP	37
9	3-*O*-α-l-Rhamnopyranosyl kaempferol 7-*O*-α-l-rhamnopyranoside	CC	39
10	3-*O*-α-l-Arabinofuranosyl kaempferol 7-*O*-α-l-rhamnopyranoside	CC	39
11	3-*O*-β-d-Apiofuranosyl-(1→2)-α-l-arabinofuranosyl kaempferol 7-*O*-α-l-rhamnopyranoside	CC	39
12	3-*O*-β-d-Glucopyranosyl-(1→3)-α-l-rhamnopyranosyl kaempferol 7-*O*-α-l-rhamnopyranoside	CC	39
13	Quercetin 3-*O*-(3′′,4′′-di-*trans-p*-coumaroyl)-α-l-rhamnopyranoside	Stem bark of CCP	125
14	Quercetin 3-*O*-(2′′,4′′-di-*trans-p*-coumaroyl)-α-l-rhamnopyranoside	Stem bark of CCP	125
15	3′′-*trans-p*-Coumaroylquercitrin	Stem bark of CCP	125
16	4′′-*trans-p*-Coumaroyl-kaempferol-3-*O*-α-l-rhamnoside	Stem bark of CCP	125
17	4′′-*cis-p*-Coumaroyl-kaempferol-3-*O*-α-l-rhamnoside	Stem bark of CCP	125
18	Kaempferol 3-*O*-(3′′,6′′-di-*trans-p*-coumaroyl)-β-d-glucopyranoside	Stem bark of CCP	125
19	Tiliroside	Stem bark of CCP	125
20	Kaempferol 3-*O*-(3′′,6′′-di-*trans-p*-coumaroyl)-β-d-galactopyranoside	Stem bark of CCP	125
21	Kaempferol 3-*O*-β-d-glucopyranoside	Stem bark of CCP	125
22	Quercetin 3-*O*-β-d-glucopyranoside	Stem bark of CCP	125
23	Quercetin 3-*O*-α-l-rhamnopyranoside	Stem bark of CCP	125
24	Quercetin 3-*O*-α-d-arabinopyranoside	Stem bark of CCP	125
25	Phenylmethanol-*O*-α-l-arabinofuranosyl (1→6)-β-d-glucopyranoside	Immature buds of CCP	137
26	Phenylmethanol-*O*-α-l-arabinopyranosyl (1→6)-β-d-glucopyranoside	Immature buds of CCP	137
27	Icariside DC	Immature buds of CCP	137
28	2-Phenylethyl-*O*-β-d-glucopyranoside	Immature buds of CCP	137
29	2-*O*-β-d-Glucosyl-(1*S*)-phenylethylene glycol	Immature buds of CCP	137
30	(2*S*)-Sutan-2-*O*-β-d-glucopyranoside	Immature buds of CCP	137
31	(2*S*)-2-Butan-2-*O*-β-d-apiofuranosyl (1→6)-glucopyranoside	Immature buds of CCP	137

Wei *et al.* extracted flavonoids from CCP leaf by the water diffusion method and determined the content of total flavonoids. The extraction rate was 0.39%, and it strongly inhibited *Staphylococcus aureus*, *Bacillus subtilis*, and *Escherichia coli*.^40^ Zou *et al.* extracted flavonoids from CC by the ultrasonic extraction method. The content of total flavonoids in stems, leaves, and fruits in the samples were 2.4177, 0.9097, and 0.1759 g L^−1^, respectively.^41^ Kun *et al.* optimized the extraction conditions of total flavonoids and found that the extraction rates of total flavonoids from CC and residue by ethanol extraction were 16.10% and 3.80%, respectively. The results were higher than those of water extraction and ultrasonic-assisted extraction.^42^ Lin *et al.* determined the best extraction process of CC flavonoids: the ratio of material to liquid was 1 : 10 (g mL^−1^), the extraction time was 1.5 h, and the extraction temperature was 80 °C.^43^

### 4.4 Polyphenols

**Fig. 5:**
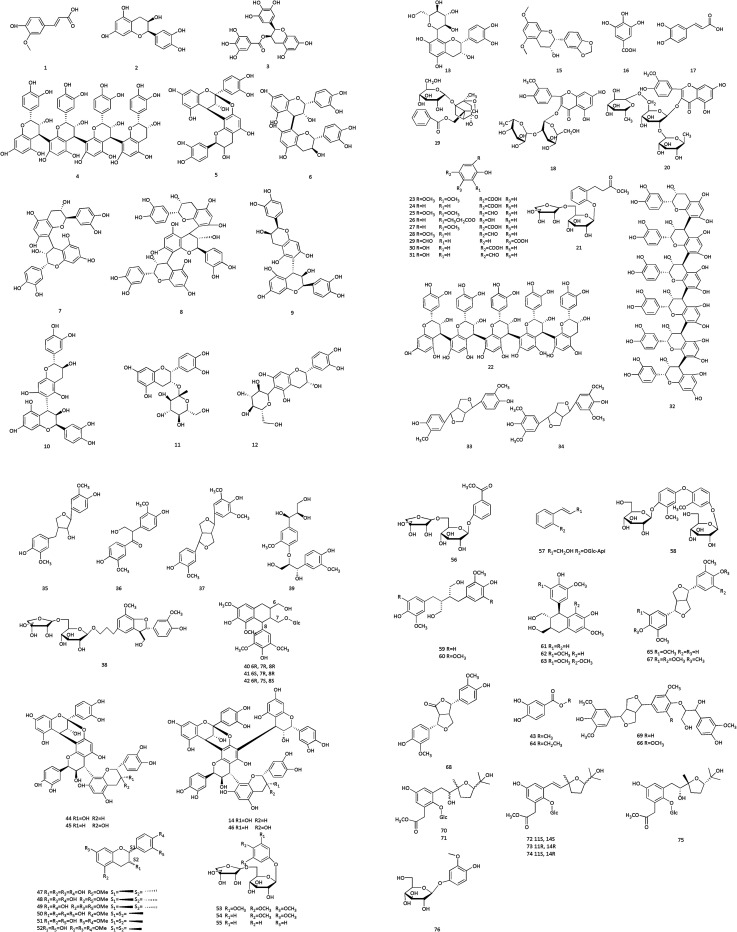
Structures of polyphenols from Cinnamomi cortex.

Polyphenols are among the principal active components of CC. They possess potent antioxidant activity and exert hypoglycemic effects. Morimoto *et al.* described three flavan-3-ol glucoside and three oligomeric procyanidins, including (−)-epicatechin 3-*O*-β-d-glucopyranoside, (−)-epicatechin 8-C-β-d-glucopyranoside, (−)-epicatechin 6-C-β-d-glucopyranoside and cinnamtannins A2, A3, and A4. There are also (−)-epicatechin, procyanidins B2, B5, C1, and others.^44^ We summarize the polyphenols in Table 6 and the structures of components in Fig. 5. Jiang *et al.* optimized the extraction process of total polyphenols from CC; the average extraction rate of total polyphenols was 3.9%.^45^

**Table 6:** Polyphenols isolated from Cinnamomi cortex

No.	Components	Sources	Ref.
1	Ferulic acid	CC	131
2	(−)-Epicatechin	CC	44
3	(−)-Epigallocatechin gallate	CC	132
4	Cinnamtannin A2	CC	44
5	Procyanidin A2	CC	133
6	Procyanidin B1	CC	133
7	Procyanidin B2	CC	44
8	Procyanidin C1	CC	44
9	Procyanidin B5	CC	44
10	Procyanidin B7	CC	134
11	(−)-Epicatechin 3-*O*-β-d-glucopyranoside	CC	44
12	(−)-Epicatechin 8-β-d-glucopyranoside	CC	44
13	(−)-Epicatechin 6-β-d-glucopyranoside	CC	44
14	Parameritannin A1	CC	119
15	(2*R*,3*R*)-5,7-Dimethoxy-3′,4′-methylenedioxy-flavan-3-ol	CC	135
16	Gallic acid	CC	131
17	Caffeic acid	CC	131
18	Isohamnetin-3-*O*-neohesperidoside	CC	131
19	Paeoniflorin	CC	131
20	Typhaneoside	CC	131
21	Cinnacasolide D	CC	27
22	Cinnamtannin A3	CC	44
23	Syringic acid	CC	119
24	4-Hydroxybenzoic acid	CC	27
25	Syringaldehyde	CC	27
26	5-Hydroxyethyl salicylate	CC	27
27	Vanillic acid	CC	131
28	Vanillin	CC	27
29	Isovanillic acid	CC	27
30	Protocatechuic acid	CC	123
31	Protocatechualdehyde	CC	123
32	Cinnamtannin A4	CC	44
33	Pinoresinol	CC	27
34	Syringaresinol	CC	136
35	Lariciresinol	CC	119
36	Evofolin B	CC	119
37	Medioresinol	CC	136
38	(7*S*,8*R*)-Dihydrodehydrodiconiferyl alcohol 9′-*O*-β-d-apiofuranosyl-(1→6)-*O*-β-d-glucopyranoside	CC	35
39	1,2,3-Propanetriol,1-[4-[(1*R*,2*R*)-2-hydroxy-2-(4-hydroxy-3-methoxyphenyl)-1-(hydroxymethyl) ethoxy]-3-methoxyphenyl]-(1*R*,2*R*)-	CC	35
40	(6*R*,7*R*,8*R*)-7a-[(β-d-Glucopyranosyl)oxy]lyoniresinol	CC	35
41	(6*S*,7*R*,8*R*)-7a-[(β-d-Glucopyranosyl)oxy]lyoniresinol	CC	35
42	(6*R*,7*S*,8*S*)-7a-[(β-D-*gluco*-Pyranosyl)oxy]lyoniresinol	CC	35
43	3,4-Dihydroxybenzoate	Stem bark of CCP	125
44	Cinnamtannin B1	CC	119
45	Cinnamtannin D1	CC	119
46	Cassiatannin A	CC	119
47	7,4′-Dimethoxyl-(+)-catechin	CC	134
48	3′-*O*-Methyl-(−)-epicatechin	CC	134
49	4′-*O*-Methyl-(+)-catchin	CC	134
50	5,7,4′-Trimethoxyl-(+)-catechin	CC	134
51	5,3′-Dimethoxyl-(−)-epicatechin	CC	134
52	5,7,3′-Trimethoxyl-(−)-epicatechin	CC	134
53	3,4,5-Trime-thoxyphenol-β-d-apiofuranosyl-(1→6)-*O*-β-d-glucopyranoside	CC	35
54	3,4-Dimethoxyphenyl-*O*-β-d-apiofuranosyl-(1→6) β-d-glucopyranoside	CC	35
55	Phenol-β-d-apiofuranosyl-(1→6)-*O*-β-D-glucopy-ranoside	CC	35
56	Cinnacasolide A	Stem bark of CCP	122
57	Cinnacasolide B	Stem bark of CCP	122
58	Cinnacasolide C	Stem bark of CCP	122
59	Secoisolariciresinol	Stem bark of CCP	125
60	5,5-Dimethoxysecoisolariciresinol	Stem bark of CCP	125
61	Isolariciresinol	Stem bark of CCP	125
62	5-Methoxy-isolariciresinol	Stem bark of CCP	125
63	Lyoniresinol	Stem bark of CCP	125
64	Ethyl-3,4-dihydroxybenzoate	Stem bark of CCP	125
65	5-Medioresinol	Stem bark of CCP	125
66	Buddlenol C	Stem bark of CCP	125
67	Yangambin	Stem bark of CCP	125
68	4-Ketopinoresinol	Stem bark of CCP	125
69	Ficusesquilignan A	Stem bark of CCP	125
70	Cinnacasside G	CC	35
71	Cinnacasside F	CC	35
72	Cinnacasside A	CC	35
73	Cinnacasside B	CC	35
74	Cinnacasside C	CC	35
75	Cinnacasside E	Immature buds of CCP	137
76	Isotachioside	Immature buds of CCP	137

### 4.5 Polysaccharides

**Fig. 6:**
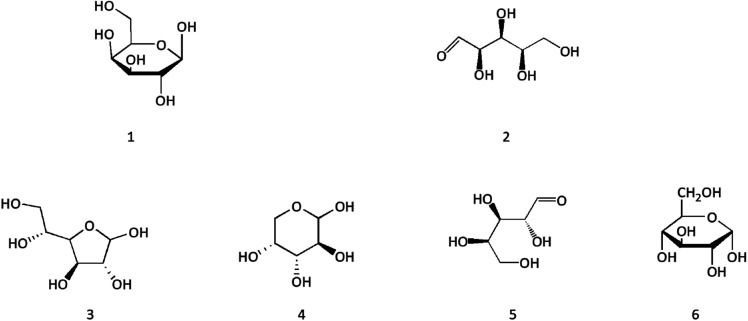
Structures of polysaccharides from Cinnamomi cortex.

Studies showed that polysaccharides in CC have antibacterial and antioxidant effects. A neutral polysaccharide (cinnaman AX) was isolated from the dried bark of CCP by Kanari M *et al.* It was composed of l-arabinose and d-xylose in a molar ratio of 4 : 3, and its molecular weight was determined to be 10^6^.^46^ Liu *et al.* compared CC and Cinnamomi Ramulus's chemical constituents and found that they all contained polysaccharides.^47^ Qin *et al.* optimized the extraction process of total polysaccharides in CC and obtained the best extraction process: the ratio of solid to liquid was 1 : 20, the extraction time was 3 h, ethanol concentration was 80%, and the extraction temperature was 80 °C.^48^ Wei *et al.* extracted polysaccharides from *Cinnamomum cassia* leaf by the water diffusion method; the amount of polysaccharide was 40.879 mg mL^−1^, and the extraction rate was 0.818%.^40^ Li *et al.* used GC-MS to analyze polysaccharide content in CC and isolated the following six compounds: galactose, d-xylose, d-glucofuranose, d-arabinose, d-ribose, and α-d-glucopyranose. The proportion of d-glucofuranose was the largest, accounting for 38.64%.^49^ The structures of polysaccharides are shown in Fig. 6.

### 4.6 Others

The ingredients in Chinese herbal medicines are complex. In addition to the ingredients summarized above, there are components with low content and some trace elements that have been. Using inductively coupled plasma mass spectrometry/inductively coupled plasma-atomic emission spectrometry (ICP-MS/ICP-AES), Chen investigated trace elements from CC, including Li, Be, Tl, Mo, Pb, Cd, Sr, V, Cr, Cu, Ni, Co, Na, B, Mg, Al, P, Ca, Ti, Zn, Ba, Mn, Fe, K. Of these, the content of Ca accounted for the highest, and Be was the lowest.^50^ CC tastes sweet and pungent. This may be related to trace elements. The correlations further study.

In this paper, the chemical constituents of CC were summarized, providing a scientific basis for studying the relationship between the biogenic pathway, traditional efficacy, modern pharmacological effects, and chemical constituents. This work creates a basis for synthesizing lead compounds, developing new drugs for clinical use, and expanding their commercialization. Nevertheless, many components in CC have not been identified, and this fact is an obstacle to its qualitative and quantitative analysis. Moreover, the research on these components focuses on an essential oil, and there are relatively few reports on polyphenols, flavonoids, and terpenoids. It is hoped that future works will concentrate on these compounds.

## 5. Analytical method and quality control

There are several components in CC; therefore, there is substantial demand for a first-rate analytical method. With the development of science, technology, and instruments, analytical methods are also improving. The methods reported in previous studies include gas chromatography, liquid chromatography, GC-MS, high-performance liquid chromatography (HPLC), thin-layer chromatography (TLC), second-derivative differential pulse polarography, ultraviolet spectrophotometry, and others. The trend is towards increasingly fast, inexpensive instruments that are environmentally friendly. HPLC is the most commonly used method. The Chinese Pharmacopoeia (2020 edition) suggests TLC and HPLC for the qualitative and quantitative analysis of indicator cinnamaldehydes, respectively. Most of these methods are rapid, sensitive, accurate, simple to operate, and have a wide application range. Their disadvantages include complex pre-treatments, expensive instruments, high costs of organic solvents, time-consuming procedures, and resources that pollute the environment. In the future, we believe that technological advances will yield cheaper, more sensitive, and environmentally friendly instruments.

Jin *et al.* suggested that the quality standard of CC should be as follows: moisture content 4.98–6.47%, total ash content not higher than 6.10%, acid-insoluble ash not higher than 3.84%, water-soluble leachates by cold soaking and hot soaking not less than 0.51% and 3.41% (respectively), and alcohol-soluble extracts by cold soaking and hot soaking, not less than 1.08% and 3.11, respectively. The essential oil content shall not be less than 3.44%, and the cinnamaldehyde content determined by HPLC should not be less than 0.25%.^51^ Li *et al.* established a method for simultaneous determination of coumarin, cinnamic acid, and cinnamaldehyde in CCP leaves using HPLC.^52^ Ma *et al.* determined the contents of cinnamaldehyde and cinnamic acid in CC from 16 areas by using HPLC and found that the contents of cinnamaldehyde and cinnamic acid in CC from Vietnam were the highest.^53^ Yuan *et al.* determined the contents of seven phenylpropanoids in CC and Cinnamomi ramulus using HPLC. They found that the content ratios of (cinnamyl alcohol + cinnamic acid)/cinnamaldehyde were 0.0121–0.0467 and 0.0598–0.182, respectively, which could be used to distinguish CC (<0.05) from Cinnamomi ramulus (>0.05).^54^

Most reports consider the content of chemical components as the basis for the quality evaluation of CC, while pharmacological activity as a quality evaluation method is rarely reported. More quality control methods based on the activity should be established.

## 6. Pharmacology

CC is a traditional Chinese medicine with many activities, including anti-inflammation, antibacterial, anti-oxidation, antitumor, and others. Various experts and scholars have confirmed these. The pharmacological effects of CC are summarized in Table 7.

**Table 7:** The pharmacological effects of Cinnamomi cortex or ingredients

Pharmacological effects	Extracts/compounds dose	Animal/cell line	Study design	Mechanism/results	Ref.
Anti-inflammatory	Cinnamaldehyde (30 mg kg^−1^), Cinnamomi cortex essential oil (30 mg kg^−1^)	CVB3-induced viral myocarditis mice model	*In vivo*	Cinnamomi cortex essential oil treated viral myocarditis that may be related to the selective inhibition of TLR4-NF-κB signal pathway by Cinnamic aldehyde	55
	Cinnamaldehyde (0.25, 0.5, 1 mg kg^−1^)	Xylene-induced auricle swelling in mice	*In vitro*	The middle and high doses of cinnamaldehyde have the inhibitory activity for the auricle swelling of mice	56
	Cinnamomi cortex-*Portulaca oleracea* (100 200 400 mg kg^−1^)	UC model mice induced by 3% DSS	*In vivo*	Treating by repairing the ulcer of colon mucosa, increasing the content of IL-10 and reducing the level of TNF-α	57
	Cinnamomi cortex extract (50 μg g^−1^ body weight)	TNBS-induced colitis mice model	*In vivo*	The protective effects of extracts against experimental colitis realized by increasing IL-10 levels, reducing the levels of IL-1β, IFN-γ, TNF-α and inhibiting the expression of COX-2	58
	*trans*-Cinnamaldehyde (50–500 μM), Cinnamomi cortex extract (1–10 μL mL^−1^)	RBL-2H3 cells or human intensive mast cells	*In vitro*	Reduced the release of β-hexosaminidase, LTC4, CXCL8, CXCL8, CCL2, CCL3, CCL4 and caused a down-regulation in ERK and PLCγ1 phosphorylation	59
Anti-bacteria	Cinnamaldehyde (1.6–6.4 mmol L^−1^, 12.8–25.6 mmol L^−1^)	*Escherichia coli*, *Staphylococcus aureus* and *Streptococcus*	*In vitro*	Cinnamaldehyde has good antibacterial activity and no drug resistance	60
	Cinnamomi cortex essential oil (0.156–10 μL mL^−1^)	*Penicillium, Aspergillus niger*	*In vitro*	The composite essential oils of Cinnamomi cortex have obvious inhibitory effect on *Penicillium* and *Aspergillus niger*	61
	Cinnamomi cortex essential oil	*Staphylococcus cremoris*, *Bacillus subtilis*, *Escherichia coli*, *Aspergillus Aspergillum niger, Penicillium* sp and *Saccharomyces cerevisiae*	*In vitro*	The Cinnamomi cortex essential oil has strong bacteriostatic action on the tested bacteria	62
	Cinnamomi cortex essential oil (1 mL)	Intestinal flora of SD rats	*In vitro*	Essential oil had a greater regulatory effect on intestinal flora	63
	Water solution and emulsion of Cinnamomi cortex essential oil	*Escherichia coli*, *Staphylococcus aureus*, *Candida albicans*	*In vitro*	Antibacterial effect of essential oil emulsion on *Staphylococcus aureus* and *Candida albicans* was weakened	64
	Petroleum ether extract of CC (LD_50_ 4.64 μg cm^−2^), ethyl acetate extract of CC (1.44 μg cm^−2^), methanol extracts extract of CC	*Dermatophagoides farinae*	*In vitro*	Petroleum ether and ethyl acetate extracts of Cinnamomi cortex had good killing activity against *Dermatophagoides farina*, while methanol extracts had no acaricidal activity	65
	Alcohol extract of Cinnamomi cortex (0.25–1 mL g^−1^)	*Escherichia coli*, *Bacillus subtilis* and *Patchouli*	*In vitro*	CC had the strongest bacteriostatic effect more than fruit and leaf of aniseed, chrysa	66
	Cinnamaldehyde (0.01–0.1 mg mL^−1^), cinnamic acid (0.25–2.5 mg mL^−1^),ethyl cinnamate (0.1–0.5 mg mL^−1^)	*Colletotrichum gloeosporioides* Penz. drug resistant strain, *Colletotrichum gloeosporioides* Penz. sensitive strain, *Banana bacterial wilt*	*In vitro*	Cinnamaldehyde had the best bacteriostatic effect, and its inhibitory effects were obviously stronger than others	67
Antioxidant	Cinnamomi cortex flavonoids (10–100 μg mL^−1^)	PC12 cell PD model induced by 6-OHDA	*In vitro*	Phenolic hydroxyl in flavone structure had reducibility and could inhibit cell membrane lipid	70
	Ethyl acetate extract, resin purification and crude extract of cinnamon tannin (0.01–0.6 mg mL^−1^)	Hydroxyl radical and DPPH radical	*In vitro*	Cinnamon tannin samples have strong scavenging ability on hydroxyl radical and DPPH radical	68
Anti-tumor	Cinnamomi cortex-Rhizoma coptidis-radix et rhizoma Rhei (0.4 mL d^−1^)	Nude mice liver cancer model	*In vivo*	Cinnamomi Cortex-Rhizoma coptidis-radix et rhizoma Rhei compound and their components compatibility can inhibit the tumor proliferation, thus benefiting blood and improving the life quality of patients	71
	Cinnamaldehyde (IC_50_ 0.36 mg mL^−1^)	Lung cancer cell line A549	*In vitro*	Cinnamaldehyde can inhibit the proliferation of human lung cancer cell line in a dose-dependent manner	72
	Cinnamaldehyde (10 μg mL^−1^)	Balb/c null female nude mice melanoma model	*In vivo*	Cinnamaldehyde can inhibit the growth of melanoma by inhibiting the expression of VEGF and HIF-α	73
	Cinnamaldehyde (0–40 μmol L^−1^)	Human melanoma cell line A375	*In vitro*	Cinnamaldehyde can inhibit the activation of NF-κB, thus inhibiting the migration and invasion of A375 cells in a dose-dependent manner	74
	Cinnamic acid (3 mmol L^−1^)	Osteosarcoma MG-63 cells	*In vitro*	Cinnamic acid can inhibit the proliferation of human osteosarcoma MG-63 cells and induce the differentiation of MG-63 cells into osteoblasts	75
	Butanol fractions extract of Cinnamomi Cortex (100 μg mL^−1^)	Human hepatocellular carcinoma cell line Hep3B	*In vitro*	Butanol fractions extract of Cinnamomi cortex have a strong inhibition on MMP-9 activity	76
Improve glucose and lipid metabolism	Powder of Cinnamomi cortex (0.1 mL 84 mg kg^−1^), water extract of Cinnamomi cortex (150–1250 μg mL^−1^)	3T3-L1 adipocytes and diabetic mouse models	*In vivo*/*in vitro*	Cinnamomi cortex water extract may be effective in the treatment of diabetes	77
	Water extract of Cinnamomi cortex (1 g mL^−1^)	Diabetic rat model	*In vivo*	Cinnamomi cortex can increase the storage of glycogen in liver and muscle of rats, so as to improve the utilization of glucose in peripheral tissues and improve insulin resistance in type 2 diabetic rats	78
	Ethanol extract of Cinnamomi cortex polysaccharide (600 mg kg^−1^)	Alloxan-induced diabetic mice	*In vivo*	Cinnamomi cortex polysaccharide with test dose could significantly reduce the blood glucose levels of the experimental diabetic mice	82
	Extract of Cinnamomi cortex polyphenol (100–300 mg kg^−1^ d^−1^), extract of Cinnamomi cortex polyphenol (1–60 μg mL^−1^)	Diabetic mice HepG2 cells	*In vitro*/*in vivo*	Cinnamomi cortex polyphenol can inhibit the activity of PEPCK, down-regulate the mRNA expression of GLUT2, PEPCK, the key rate-limiting enzyme in gluconeogenesis metabolic pathway and G6P	81
	Water extract of Cinnamomi cortex (50–200 mg kg^−1^)	Type II diabetic mice model (C57BIKsj db/db)	*In vivo*	The hypoglycemic effect of Cinnamomi cortex could be realized by improving insulin sensitivity and reducing the absorption of carbohydrates in the small intestine	80
	Cinnamomi cortex polyphenol (1–100 μg mL^−1^)	HpeG2 cells	*In vitro*	Cinnamomi cortex polyphenol can improve lipid deposition in hepatocytes by inhibiting lipid *de novo* synthesis through SIRT1-AMPK-ACC pathway	79
Neuroprotection	Aqueous extract of Cinnamomi cortex (100 μg mL^−1^)	AD drosophila and AD transgenic mice	*In vivo*	Extract markedly inhibits the formation of toxic Ab oligomers and prevents the toxicity of Ab on neuronal PC12 cells	83
	Powder of Cinnamomi cortex (200 mg kg^−1^ body wt d^−1^), Metabolite sodium benzoate (50–1000 μM)	Mice	*In vivo*	Sodium benzoate could, through PKA-CREB pathway, up-regulate the levels of brain-derived neurotrophic factor (BDNF) and neurotrophin-3 (NT-3) in the central nervous system of mice	84
	Procyanidin type-A trimer (0.01–0.1 mg mL^−1^), cinnamaldehyde (10–50 μM), coumarin (10–50 μM)	Glial cell swelling induced by glucose and oxygen deprivation	*In vitro*	Trimer 1 had the ability to inhibit oxygen free radical content and calcium movement to reduce nerve cell swelling in ischemic injury, and also had a regulatory role by preventing the decline in glutamate uptake to reduce glutamate excitotoxicity	85
Others	Cinnamaldehyde (75 mg kg^−1^ d^−1^)	Osteoporosis rats induced by ovariectomy	*In vivo*	Cinnamaldehyde could significantly increase BMD, trabecular number, trabecular thickness, trabecular area and reduce trabecular separation and serum TNF-α and IL-6 levels	86
	Cinnamon essential oil (60 μL L^−1^)	Dangshan pears	*In vitro*	Cinnamomi cortex essential oil has a good fresh-keeping effect on postharvest Dangshan pear.	87
	Ethanol extract of *Cinnamomum cassia* (L.) J. Presl leaves (0.391–100 μM)	ConA-induced mouse T lymphocytes	*In vivo*	Reduce inflammation factors including IL-1β, IL-6, TNF-α, PGE 2 levels and adjust the hypothalamus cAMP and the synthesis of AVP secretion	88
	Aqueous extract of Cinnamomi cortex (200 mg mL^−1^)	ADP-induced platelet aggregation in rats	*In vitro*	Cinnamomi cortex inhibits platelet aggregation induced by VADP in rats and has anticoagulant effect *in vitro*, suggesting that Cinnamon can prevent venous or arterial thrombosis	89
	Cinnamomi cortex (1.8 g kg^−1^)	Adrenal regeneration hypertensive rat model	*In vivo*	Cinnamomi cortex can significantly reduce blood pressure (*p* < 0.001) and urinary aldosterone excretion (*p* < 0.001), and significantly increase the content of enkephalin in striatum and hypothalamus (*p* < 0.001)	90

### 6.1 Anti-inflammatory effects

CC can be used to treat diseases caused by inflammatory factors. Viral myocarditis is frequently seen in the clinic. It is caused by the heart being infected by viruses, resulting in localized or diffuse myocarditis. CC is a highly effective treatment for viral myocarditis. Ding *et al.* investigated the pharmacological mechanisms of essential oils on coxsackievirus B3 (CVB3)-induced viral myocarditis (VMC) in mice. They found that the mortality of mice treated with essential oil or cinnamaldehyde (CA) was significantly lower, and the median survival time was prolonged. The expression of inducible nitric oxide synthase, tumor necrosis factor (TNF-α), nuclear transcription factor kappa B (NF-κB), P65, and Toll-like receptor 4 protein in myocardium decreased significantly (*P* < 0.05). There was no statistical difference between the two treatment groups (*P* > 0.05). Experiments confirmed that the mechanism of essential oil in treating VMC might be related to CA, which selectively inhibits TLR4-NF-κB signaling *in vivo*. This study provides a scientific basis for clinical treatment in VMC using essential oil.^55^ However, the experiment only investigated a single dose of essential oil and cinnamaldehyde at 30 mg kg^−1^ and lacked analysis of maximum and minimum effective doses. In addition, only cinnamaldehyde was selected as the indicator control, and there was no positive control group, compromising the validity of the findings. The components in CC are complex, and the anti-inflammatory effects of other components should be further studied.

Hu *et al.* studied the anti-inflammatory effect of cinnamaldehyde on xylene-induced auricle swelling in mice. The results showed that middle and high doses of cinnamaldehyde significantly inhibited auricle swelling of mice, suggesting that it inhibits acute inflammation. The effect was significantly better than that of aspirin and low-dose cinnamaldehyde (*P* < 0.05).^56^ However, this study only investigated an animal model *in vitro* and lacked analyses at the cellular and clinical practice levels. Meanwhile, detail anti-inflammatory mechanisms and targeted pathways need to be explored further in this study.

Ren *et al.* evaluated the anti-inflammatory activity of CC-*Portulaca oleracea* on an ulcerative colitis (UC) model in mice induced by 3% dextran sodium sulfate. The authors found that ulcers of the colon mucosa in mice in the administration group were somewhat repaired. Unlike the model group, the content of serum anti-inflammatory factor interleukin (IL)-10 increased significantly (*P* < 0.01), and the content of pro-inflammatory factor TNF-α decreased significantly (*P* < 0.01), suggesting that the combination of the herb pair inhibited UC activity. This experiment provides a new basis for the treatment of UC.^57^ Unfortunately, the method was tested only at the animal level. Its therapeutic mechanism at the cellular and molecular levels should be studied in the future. The specific active compounds that mediate the anti-inflammatory role should also be studied to provide a basis for developing effective and safe drugs with few side effects.

Kwon *et al.* found that the anti-inflammatory mechanisms of CC extract in a 2,4,6-trinitrobenzenesulfonic acid-induced colitis mice model were related to regulatory dendritic cells (RDCs). RDCs produced low levels of the pro-inflammatory cytokines IL-1β, interferon-gamma, and TNF-α, and high levels of the immunoregulatory cytokine IL-10 after medication. Treatment was also associated with the inhibition of cyclooxygenase-2.^58^ However, this experiment was confined to cellular and protein related to anti-inflammation and did not fully explore the mechanistic pathways. There are many components in CC extract; the components with the primary anti-inflammatory effect and the structural material bases of this effect need to be studied.

Hagenlocher *et al.* analyzed the effect of cinnamaldehyde, the active ingredient of CC, on the activation of mast cells. They found that cinnamaldehyde reduced the release of β-hexosaminidase in human-intensive mast cells (hiMC) and RBL-2H3 cells. Expression of LTC4, CXCL8, CCL2, CCL3, and CCL4 was significantly inhibited in hiMC by cinnamaldehyde. The phosphorylation of extracellular signal-regulated kinase and phospholipase γ1 was inhibited. The outcomes were similar to CC extract, suggesting that cinnamaldehyde is the active component mediating CC's anti-inflammatory and anti-allergic properties.^59^ However, this experiment did not study the relationship between the chemical structure of cinnamaldehyde and its pharmacological activity. The bioavailability, activity at the cellular level, and whether its mechanism proceeds successfully should be further study subjects.

### 6.2 Anti-bacterial effects

Cheng *et al.* studied the antibacterial effect of cinnamaldehyde and tested its antibacterial effect on three pathogenic bacteria that cause mastitis in dairy cows using the double dilution method. They found that the minimum inhibitory concentration (MIC) of cinnamaldehyde against *E coli*, standard *E. coli*, *S. aureus*, standard *S. aureus,* and *Streptococcus in vitro* was 6.4 mmol L^−1^, 3.2 mmol L^−1^, 3.2 mmol L^−1^, 1.6 mmol L^−1^, and 1.6 mmol L^−1^, respectively. The minimum bactericidal concentrations (MBCs) were 25.6 mmol L^−1^, 12.8 mmol L^−1^, 25.6 mmol L^−1^, 12.8 mmol L^−1^, and 12.8 mmol L^−1^, respectively.^60^ These findings suggested that cinnamaldehyde is a potential clinical treatment for bovine mastitis; furthermore, according to the standard of bacterial drug resistance (MBC ≥ 32 MIC), there was no drug resistance. Nevertheless, the investigators only compared the inhibitory effects of cinnamaldehyde on several bacteria, and the mechanism of the inhibition was not studied; furthermore, the reasons for the differences in inhibition rates among the three bacteria were not explored. There were differences between the bacteriostatic effect *in vitro* and *in vivo* (in cows). We suggest conducting experiments in animals and measuring the production of drug-resistant bacteria.

Xu *et al.* found that essential oil significantly inhibited *Penicillium* and *Aspergillus niger*, common pathogens in winter jujube, with MICs of 0.313 mmol L^−1^ and 0.156 mmol L^−1^, respectively. These findings suggest that the oil is a suitable inhibitor of winter jujube storage mold.^61^ Gu *et al.* studied the antibacterial activity of CC essential oil against six bacteria, including *Staphylococcus cremoris*, *Bacillus subtilis*, *E. coli*, *Aspergillus niger*, *Penicillium* sp*.* and *Saccharomyces cerevisiae*. They found that essential oil potently inhibited bacteria, mold, and yeast, in the following order mold > yeast > bacteria. The bacteriostatic effect was affected by pH, with the optimal range of pH 3–7. The bacteriostatic effect decreased with increasing pH.^62^ The antibacterial mechanism *in vivo* was not studied, and there was no MIC or positive control. Therefore, the reliability of these results is in doubt.

Liang *et al.* measured the inhibitory effect of CC essential oil on the growth of intestinal bacteria in rats and found that most of the intestinal bacteria were inhibited; however, the growth of lactic acid bacteria was not affected, suggesting that essential oil had a greater regulatory effect on intestinal flora.^63^ An in-depth study on the morphological structure of lactobacillus may reveal the mechanism of drug resistance. This experiment had no positive control group, although there was a blank control.

Huang *et al.* studied the changes of antibacterial effect when the essential oil was emulsified. They found that the antibacterial effect of the emulsion on *E. coli* was the same as that of the aqueous solution group; however, its antibacterial effect on *S. aureus* and *Candida albicans* was weakened, possibly related to the different structures and morphologies among Gram-negative bacteria, Gram-positive bacteria and fungi.^64^ Nevertheless, there was no investigation of the mechanism of bacteriostasis. There was no positive control group and no measurement of the maximum bacteriostatic concentration.

Li *et al.* measured the killing activity of different solvent extracts of CC against *Dermatophagoides farinae* and found that CC's petroleum ether and ethyl acetate extracts had good killing activity against the organism. The lethal dose 50s (LD_50_s) were 4.64 μg cm^−2^ and 1.44 μg cm^−2^, respectively, in a concentration- and time-dependent manner. When the dosages reached 6.09 μg cm^−2^ and 9.75 μg cm^−2^, the acaricidal rate was 100% after 24 h, while methanol extracts had no acaricidal activity.^65^ Chen *et al.* found that the alcoholic extracts of CC and aniseed had sound inhibitory effects on *E. coli*, *B. subtilis*, and *Patchouli*. CC had a more substantial bacteriostatic effect more than aniseed fruit and leaf (*p* < 0.05). This study suggested that the inhibitory effect of CC on *B. subtilis* was better than that of the preservative chrysa, suggesting that CC has the potential to be utilized as a preservative.^66^ However, the authors did not study the bactericidal chemical constituents that dominated the role of CC. Moreover, the pharmacological mechanism of inhibiting CC remained at the experimental phenomenon level. Future research should explore the drug targets and mechanisms.

Guo *et al.* compared the bacteriostatic effects of cinnamaldehyde, cinnamic acid, and ethyl cinnamate. They found that the cinnamaldehyde has the best bacteriostatic effect, even better than traditional fungicide carbendazim, with the potential to be used as a pollution-free bactericide.^67^

### 6.3 Antioxidant effects

Chen *et al.* found that three cinnamon tannin samples had strong scavenging ability on hydroxyl radical and 2,2-diphenyl-1-picrylhydrazyl (DPPH) radical in a concentration-dependent manner. When the concentrations of ethyl acetate extract, resin purification, and crude extract were 0.04 mg mL^−1^, the clearance rates of DPPH radical were 90.98%, 90.48%, and 89.38%, respectively. These rates were higher than the clearance rates of ascorbic acid at the same concentration of 50.2%. For hydroxyl radical, the maximum clearance rates of the three samples were all over 65% and increased in a dose-dependent manner in the range of 0.1–0.6 mg mL^−1^. CC tannin can be developed as a natural antioxidant in the future. This study had profound and extensive significance.^68^ Nevertheless, a limitation of the study was that it only included *in vitro* experiments; the bioavailability and antioxidant capacity of tannins in humans are not clear. Studying only the scavenging capacity of hydroxyl and DPPH radicals cannot reflect the total antioxidant capacity of tannins.

Li *et al.* analyzed the total antioxidant properties of CC essential oil and found that essential oil was weaker in antioxidant capacity, inhibition of lipid peroxidation in linoleic acid, and scavenging DPPH radical hydroxytoluene (BHT) and propyl gallate (PG). In terms of scavenging hydroxyl radical, the essential oil group was the strongest, followed by BHT, and PG was the weakest. The essential oil was more potent than PG at 1.0 mg mL^−1^.^69^ The authors concluded that CC essential oil was superior to synthetic antioxidants in some aspects and might be a candidate for use in the food industry. Nevertheless, it remained unclear whether the antioxidant activity of essential oils was the result of a component or a combination of several components; therefore, it is necessary to screen them in detail. There is also a problem about whether its antioxidant performance changes *in vivo*. The relationship between its basic structure and pharmacological activity is also worth studying.

Flavonoids from CC have good antioxidant effects. Zeng *et al.* studied the pharmacological action and mechanism of CC flavonoids on a Parkinson's disease model established by 6-hydroxydopamine (6-OHDA)-induced injury to PC12 cells. The cell survival rate and superoxide dismutase activity were significantly increased (*P* < 0.05). The apoptosis rate, DNA damage, Bax/Bcl-2 ratio, caspase-9 expression, and malondialdehyde content was significantly lower; these effects were dose-dependent. These findings suggest that total flavonoids of CC protect mitochondria by inhibiting oxidative stress, thereby reducing the damage of 6-OHDA to PC12 cells.^70^ However, the study was conducted only at the cellular level. Medications are affected by several factors after entering the body. Therefore, it is best to test animal models and verify the clinical level results to develop safe and effective medications from natural antioxidants.

### 6.4 Anti-tumor effects

Tumors are characterized by mutated cells growing irregularly and rapidly due to gene mutations or DNA replication errors. Tumors can be divided into benign and malignant classes. Malignant tumors can be further divided into carcinoma and sarcoma according to the site of origin. Cancer is one of the three diseases with the largest death in the world. Chemotherapy is the primary treatment; however, these treatments are expensive and carry substantial side effects. CC invigorates qi and keeps the individual healthy, improving immunity and resistance to cancers.

Qian *et al.* studied the antitumor effect of the *rougui* (CC)-Huanglian (Rhizoma coptidis)-Dahuang (Radix et Rhizoma Rhei) herb pair on a nude mice liver cancer model induced by injection of human liver cancer cells. The tumor weights in the compound and berberine–emodin–cinnamaldehyde groups were lower than the model group, suggesting that both inhibited tumor proliferation, possibly by improving qi and blood and improving quality of life.^71^ Although some achievements were made in this experiment, there was no comparison with current anticancer drugs; therefore, the anticancer effect is unclear and should be evaluated. Although the study showed that the compound and groups inhibited tumor proliferation, the maximum safe doses were not determined. Finally, the mechanism of action was not explored.

Song *et al.* added cinnamaldehyde to the lung cancer cell line A549 and studied its inhibitory effect on cell growth. They found that the cells generated vacuoles, swelling, and shedding after treatment, suggesting that cinnamaldehyde had good antitumor activity and inhibited the proliferation of lung cancer cells in a dose-dependent manner, with an IC_50_ of 0.36 mg mL^−1^.^72^ Seo *et al.* screened for anticancer effects in herbs with matrix metalloprotease-9 inhibitory activity from 87 herbal extracts and found that the inhibitory activity of cinnamyl butanol extract at the concentration of 100 μg mL^−1^ was over 90%.^76^ Li *et al.* studied the antitumor effect of cinnamaldehyde in a Balb/c null female nude mice melanoma model and found that the melanoma tumor volume and the number of new blood vessels in the cinnamaldehyde treatment group were significantly lower. Expression of vascular endothelial growth factor and hypoxia-inducible factor was inhibited, suggesting possible anti-melanoma mechanisms.^73^ However, there were no positive controls, and the antitumor mechanism of cinnamaldehyde was not studied in depth. Although two tumor-related factors were confirmed, the inhibition mechanism remains unclear. The lowest effective and safe dose of cinnamaldehyde should also be investigated to provide the basis for rational treatment.

Zhou *et al.* studied the effect of cinnamaldehyde on the invasion ability of human melanoma cell line A375 *in vitro* and found that cinnamaldehyde inhibited the migration and invasion of A375 cells in a dose-dependent manner. The mechanism of action may be inhibition of NF-κB activation.^74^ Wang *et al.* studied the pharmacological effects of cinnamic acid on a differentiation model of osteosarcoma MG-63 cells *in vitro* and found that the ratio of G0/G1 cells in the cinnamic acid group was significantly greater. The morphology and ultrastructure of MG-63 cells returned to normal. The expression of osteoblast differentiation markers I collagen, osteomucin, and osteocalcin increased, and calcium deposition and formation of typical bone joints accelerated, suggesting that cinnamic acid inhibited the proliferation of MG-63 cells and induced them to differentiate into osteoblasts.^75^ However, these experiments were confined to a cultured cell model, and the pharmacological mechanism obtained from these experiments was unclear. The optimal antitumor dose was not determined. The inhibitory process of cinnamaldehyde or cinnamic acid on tumors is complex, involving many enzymes, protein factors, and pathways. The mechanisms obtained in mentioned experiments did not explain the entire antitumor process. Future work might exploit network pharmacology to predict mechanisms of action and verify them to improve the antitumor effect of CC.

### 6.5 Improvement of glucose and lipid metabolism

Diabetes is characterized by high blood sugar levels caused by insufficient insulin secretion or insulin resistance (types 1 and 2, respectively). Type 2 is more common and is the result of genetic and environmental factors. If diabetes is not controlled in a timely fashion, it causes kidney, nerve, eye, foot, heart, and brain complications. In short, diabetes is among the major diseases endangering human health worldwide. Its incidence increases yearly. At present, western medicine is the primary method of treatment; nevertheless, exacerbations are common. CC has a mild and lasting hypoglycemic effect, attracting substantial attention in China and abroad.

Xiang *et al.* studied the hypoglycemic effects of CC powder and water extracts on 3T3-L1 adipocytes and diabetic mice and found that CC coarse powder did not affect 3T3-L1 adipocytes. The water extract of CC promoted fat droplet growth at 750 μg mL^−1^ and inhibited the proliferation of 3T3-L1 adipocytes at 156–1250 μg mL^−1^. The inhibitory mechanism at 1250 μg mL^−1^ might involve regulation of peroxisome proliferator activated receptor γ (PPAR-γ) and cyclic adenosine monophosphate-response element binding protein genes. The water extracts of CC at 300 mg kg^−1^ and 150 mg kg^−1^ improved blood sugar levels in mice, with insulin-like effects at 2.5 mg mL^−1^ and 5 mg mL^−1^. This experiment preliminarily suggested that CC water extract may treat diabetes.^77^

Xu *et al.* established a diabetic rat model by injecting streptozotocin and feeding a high-calorie diet. After two weeks of treatment with CC, liver, and muscle glycogen levels in the CC-treated group were significantly higher than the model group. These findings suggest that CC increases glycogen storage in the liver and muscle of rats, improving the utilization of glucose in peripheral tissues and reversing insulin resistance in type 2 diabetic rats.^78^ However, the study used only a single dose and lacked an analysis of a detailed mechanism.

Zheng *et al.* studied the biological mechanisms of CC polyphenol on lipid metabolism of HpeG2 cells at the molecular level and found that CC polyphenol reduced the level of triglycerides and downregulated sterol regulatory element-binding protein-1 (SREBP-1), a key transcription factor in the *de novo* synthesis of fatty acids; it also caused a significant reduction in mRNA expression of downstream target genes fatty acid synthase (FAS) and stearoyl-CoA desaturase 1 (SCD1). After the NAD-dependent deacetylase sirtuin-1 (SIRT1) gene became silent, the downstream pathway proteins AMP-activated protein kinase (AMPK), acetyl-CoA carboxylase (ACC), and phosphorylation levels decreased significantly. The mRNA expressions of SREBP-1, FAS, and SCD1 were significantly increased, suggesting that CC decreased lipid deposition in hepatocytes by inhibiting lipid *de novo* synthesis through the SIRT1-AMPK-ACC pathway.^79^ However, this method was not verified in clinical experiments or animal models, and there was no positive control group. Polyphenol is not the main component of CC; therefore, the mechanism by which CC polyphenol improves fat deposition in liver cells remains unclear.

Sung *et al.* found that the water extract of CC significantly decreased blood sugar levels in a type II diabetic mice model (C57BIKsj db/db) in a dose-dependent manner at 50–200 mg kg^−1^. This extract significantly increased serum insulin and high-density lipoprotein levels after 6 weeks of treatment, suggesting that the hypoglycemic effect of CC might be mediated by improving insulin sensitivity and reducing the absorption of carbohydrates in the small intestine.^80^ However, only the water extract of CC was studied without determining which components played a major role. The mechanism of CC's reversal of insulin resistance was not elaborated in depth.

Lu *et al.* found that CC polyphenol significantly reduced blood sugar levels in insulin-resistant diabetic mice but had no effect on weight. At the cellular level, the authors found that CC polyphenol did not inhibit cell growth at 1–15 μg mL^−1^. At 15 μg mL^−1^, CC polyphenol significantly increased glucokinase activity in insulin-resistant HepG2 cells. At 10 and 15 μg mL^−1^, CC polyphenol inhibited the activity of phosphoenolpyruvate carboxykinase (PEPCK). CC polyphenol inhibited the mRNA expression of recombinant glucose transporter 2 and PEPCK, the key rate-limiting enzyme in the gluconeogenesis metabolic pathway and glucose-6-phosphatase. In short, CC reversed insulin resistance by reducing the production of endogenous glucose in cells and promoting the utilization of glucose in cells.^81^ However, this experiment did not explain how CC polyphenol reversed insulin resistance, nor were there clinical experiments.

Yu *et al.* studied the hypoglycemic effect of CC polysaccharide extract on alloxan-induced diabetic mice and found no significant difference in body weight or organ coefficient between CC polysaccharide-treated and normal mice. This finding suggests that CC possesses a significant hypoglycemic effect.^82^ However, the polysaccharide extract in this experimental method was impure, affecting the accuracy of the dosage. The study was only conducted using a single dose, and there was no study of safe dose or maximum therapeutic dose.

### 6.6 Neuroprotective effects

Frydman-Marom *et al.* studied the therapeutic effect of CC extract on Alzheimer's disease (AD) drosophila and AD transgenic mice. They found that the life of AD drosophila was prolonged, exercise ability was restored, and the toxic Aβ oligomer in the drosophila brain was eliminated. After taking the CC extract, AD transgenic mice showed reduced 56 kDa Aβ oligomers and plaques and improved cognitive activity. The results suggest that CC extract prevents the toxic effects on PC12 cells by inhibiting the formation of toxic Aβ oligomers. CC might be developed into a medication that is easy to administer and can prevent and treat Alzheimer's disease.^83^ Jana *et al.* demonstrated for the first time the mechanism by which the CC metabolite sodium benzoate treats neurodegenerative diseases. *Via* the PKA-CREB pathway, sodium benzoate increased levels of brain-derived neurotrophic factor and neurotrophin-3 in the central nervous system of mice in a dose-dependent manner. These findings suggest that CC might be used as the main or auxiliary therapy for neurodegenerative diseases.^84^

Panickar *et al.* found that procyanidin type-a trimer (trimer 1), cinnamaldehyde, and coumarin isolated from CC aqueous extract blocked increases in glial cell swelling induced by glucose and oxygen deprivation. Trimer 1 inhibited oxygen-free radical content and calcium movement to reduce nerve cell swelling in ischemic injury. It also prevented the decline in glutamate uptake to reduce glutamate excitotoxicity. The authors concluded that CC could treat ischemic injury and other neurological diseases.^85^

### 6.7 Other effects

Du *et al.* found that cinnamaldehyde significantly increased bone mineral density (BMD), trabecular number, trabecular thickness, and trabecular area of osteoporosis rats induced by ovariectomy, and reduce trabecular separation, serum TNF-α, and interleukin-6 (IL-6) levels.^86^ However, this method was not verified in patients. Gou *et al.* found that the preservative effect of CC essential oil on Dangshan pears was highest at 60 μL L^−1^ when combined with 1% chitosan and 1% CaCl_2_.^87^ Zhou *et al.* found that six compounds extracted from ethanol extract of *Cinnamomum cassia* leaves boosted ConA-induced proliferation of mouse T lymphocytes by 78%. Two compounds promoted the proliferation of lymphocytes when the concentration was lower than 25 μM and inhibited the proliferation of lymphocytes when the concentration was 100 μM, suggesting that CC leaves were immunomodulatory.^88^ Chen *et al.* found that CC extract had a 72% inhibition rate on ADP-induced platelet aggregation in rats and prevented arterial thrombosis. Intravenous injection of CC significantly increased blood flow in coronary and brain arteries, possibly related to myocardial inhibition. Peripheral circulation experiments showed that CC directly dilated peripheral blood vessels.^89^ Kuang *et al.* found that CC significantly reduced blood pressure, urinary aldosterone excretion, and significantly increased enkephalin content in the striatum and hypothalamus, suggesting that CC modulates renal vascular hypertension.^90^

CC treats digestive tract ulcers, tranquilizes, relieves spasms, relieves fever, expels insects, relieves cough, eliminates phlegm, relieves asthma, treats senile nocturia, resists prostatic hyperplasia, has a hypnotizing effect.

## 7. Pharmacokinetics

Yang *et al.* studied the pharmacokinetics of cinnamic acid in Bao Xin Wei pill in rats. The regression equation between peak area (*y*) and concentration (*x*) of cinnamic acid was *Y* = 4973.5348 + 42 867.96678*x*, and the correlation coefficient was *r* = 0.9998. The recovery of cinnamic acid in plasma was 97.5%, with an relative standard deviation (RSD) of 1.33%. Oral absorption of cinnamic acid in rats was accorded with the open room model of first-order absorption and first-order elimination, with *t*_1/2_(Ka) = 7.12 min, *T*_max_ = 53.29 min, *C*_max_ = 0.20 μg mL^−1^, and *t*_1/2_(*K*_a_) = 340.74 min. The components in the compound prescription are complex, and other components can be oxidized into cinnamic acid *in vivo* except for storax and cinnamic acid in CC; these findings suggest that the pharmacokinetic parameters obtained from the experiment are comprehensive.^91^

Feng *et al.* studied the pharmacokinetics of cinnamic acid in mice. The standard curve of cinnamic acid in plasma was *y* = 0.2340*c* + 05174 (*n* = 11). The linear range was 2.95–295 μg mL^−1^, *r* = 09992. The lowest quantitative plasma concentration of cinnamic acid was 2.95 μg mL^−1^. With the signal-to-noise ratio of 3 : 1, the lowest detection concentration of cinnamic acid in plasma was 0.5 μg mL^−1^, and the recovery and RSD of drugs in plasma at three concentrations were 99.2% and 4.6%, 108% and 4.0%, and 87.5% and 6.8%, respectively. The RSDs of intra-day precision were 6.8%, 4.0%, and 4.6%, respectively. The RSDs of inter-day precision were 1.0%, 3.9%, and 11% respectively. The process of cinnamic acid in mice accorded with the two-compartment model, and the pharmacokinetic parameters of cinnamic acid in mice after intravenous injection were as follows. *A* (μg mL^−1^): 215.64; *B* (μg mL^−1^): 8.19; *α* (h^−1^): 9.17; *β* (h^−1^): 0.88; *V*(*c*) (L kg^−1^): 07; *T*_1/2(*α*)_ (h): 0.076; *T*_1/2(*β*)_ (h): 0.79; *K*_10_ (h^−1^): 6.82; area under curve (AUC) (h μg mL^−1^): 38.19; CL (L h^−1^ kg^−1^): 4.78. The pharmacokinetic parameters of cinnamic acid in mice were *A* (μg mL^−1^): 172.52; *B* (μg mL^−1^): 18.22; *α* (h^−1^): 1.69; *β* (h^−1^): 0.80; *K*_10_ (h^−1^):1.52; *K*_a_ (h^−1^): 16.54; *T*_1/2(*α*)_ (h): 0.41; *T*_1/2(*β*)_ (h): 0.87; AUC (h μg mL^−1^): 105.06; CL (L h^−1^ kg^−1^): 1.35; *T*_max_ (h): 0.l6; *C*_max_ (μg mL^−1^): 134.19. The absolute bioavailability (f) was 95.98%.^92^

Li *et al.* studied the pharmacokinetics of cinnamic acid in rabbit serum after oral administration of CC, cinnamic acid, and Jingui Shenqi Pill. The bioavailability of cinnamic acid in the CC group was 66.12% of cinnamic acid, and that of the Jingui Shenqi Pill group was 73.83% of cinnamic acid. Acidified ether was the best solvent for extracting cinnamic acid from serum. By analyzing the kinetic parameters, the authors determined that the best models were two-compartment models. The pharmacokinetic parameters of the Jingui Shenqi Pill group were quite different from the other two groups, which may relate to the compatibility of components in the prescription.^93^ Su *et al.* studied the pharmacokinetics of cinnamaldehyde in rats with deficiency heat, cold deficiency, and normal, and found that the cold deficiency group absorbed less cinnamaldehyde, distributed less cinnamic acid *in vivo*, stayed for a shorter time, and eliminated faster. This may be related to the change of metabolic enzyme activity. The specific mechanism of the pharmacokinetic difference requires further study.^94^

## 8. Conclusion and discussion

CC is an excellent natural herbal resource with characteristics that are not shared with other traditional Chinese medicines and can be used as medicine and food. This paper comprehensively illustrated the characteristics of CC in botany, traditional applications, and pharmacokinetics. It is valuable that the structure of more than 300 components isolated from CC has been summarized on paper. The investigations of anti-inflammatory, anti-oxidation, antitumor, antibacterial, hypoglycemic, neuroprotective, and other aspects of CC are also exemplified in the pharmacological section. Such in-depth and comprehensive summaries have never been reported in previous literature. Nevertheless, despite the many successes, we found there are yet many shortcomings in the study of CC.

First, limited to the current instruments and analytical methods, many components have not been discovered in CC that may be the keys to its efficacy. We recommend further research on the active components. Their development and utilization of CC should be carried out in the future. At present, most studies concern the components of CC. To better use CC resources, we suggest that more analysis on twigs, leaves, and fruits be done to widen the development of multi-active drugs.

Second, CC has a wide range of pharmacological effects. Essential oils have anti-inflammatory, antioxidative, antibacterial, and antitumor effects. Flavonoids compounds of CC exhibit good antioxidant potential. Phenolic compounds improve glucose and lipid metabolism. However, the specific mechanism of CC in humans has not been uniformly demonstrated. There are several opinions regarding the targets and pathway of drug action, and there is no consensus. It is hoped that future investigators will systematically study the specific mechanisms of various pharmacological effects of CC to improve its theoretical basis. The active compounds that can be developed and related to drug efficacy primarily concerned essential oil components, while there is less research on flavonoids and polyphenols. We suggest that more attention should be paid to flavonoids and polyphenols in the future.

Third, CC resources are abundant in China; however, there was no comprehensive resource investigation or analysis of CCP species for a long time. People randomly denominated CC according to its origin and color, causing confusion in the system and hindering the screening and development of excellent CCP strains. Therefore, checking and standardizing the species and strains of CCP is the fundamental premise for preserving and utilizing germplasm resources, and it is also an essential basis for screening excellent strains and genetic breeding of CCP in the future.

Fourth, herbal processing technology has a long history in China. These technologies enhance the efficacy and purify and detoxify herbs. However, at present, the processing of CC is based on simple mashing, and no other processing methods have been reported. Therefore, it is critical to study the processed products of CC.

Fifth, according to the 2020 edition of the Pharmacopoeia, cinnamaldehyde is the active marker of CC. However, it is unwise to cover all herbal medicine with only one marker. Future research should focus on phytochemistry, using new instruments and technologies to identify new biochemical markers and study the relationship between material infrastructure and pharmacological effects. Doing so will provide a scientific basis for future clinical development of targeted preparations.

Finally, there is little research on traditional applications. China has accumulated experience with clinical uses of CC for thousands of years. CC should be widely used currently. However, the modern pharmacological uses of CC are far less abundant than those of traditional uses. Most people only use it as a spice, possibly due to insufficient popularization of Chinese medicine knowledge. We believe more people should pay attention to cosmetic, antioxidative, antitumor effects, weight loss, and hypoglycemic effects. In short, many critical traditional applications have been neglected. To better utilize CC resources, we should conduct a comprehensive study on its application to benefit humanity.

This paper reviewed CC from botany, ethnopharmacology, phytochemistry, and pharmacology, hoping to provide a basis for future research and promote active lead compounds and innovative drugs. With the development of science and technology and the deepening of research, investigators will have a more thorough understanding of CC, and CC preparations will spread out from the Chinese market and be accepted by the entire world.

## Conflicts of interest

The authors declare no conflict of interest.

## Supplementary Material
